# Risk factors for delayed colorectal postpolypectomy bleeding: a meta-analysis

**DOI:** 10.1186/s12876-024-03251-6

**Published:** 2024-05-14

**Authors:** Xuzhen Zhang, Xiaoxing Jiang, Liang Shi

**Affiliations:** 1 Department of Gastrointestinal Endoscopy Center, Beijing Jingmei Group General Hospital, Beijing, China; 2 The Second Department of Internal Medicine, Huaping Hospital of Traditional Chinese Medicine, Lijiang City, Yunnan Province China; 3 The First Department of General Surgery, Cangzhou Central Hospital of Hebei Province, Cangzhou, Hebei Province China

**Keywords:** Colorectal polyps, Postoperative bleeding, Risk factors, Meta-analysis

## Abstract

**Background:**

To systematically analyze risk factors for delayed postpolypectomy bleeding (DPPB) in colorectum.

**Methods:**

We searched seven large databases from inception to July 2022 to identify studies that investigated risk factors for DPPB. The effect sizes were expressed by relative risk (RR) and 95% confidence interval (95% CI). The heterogeneity was analyzed by calculating *I*^*2*^ values and performing sensitivity analyses.

**Results:**

A total of 15 articles involving 24,074 subjects were included in the study. The incidence of DPPB was found to be 0.02% (95% CI, 0.01–0.03), with an *I*^*2*^ value of 98%. Our analysis revealed that male sex (RR = 1.64), history of hypertension (RR = 1.54), anticoagulation (RR = 4.04), polyp size (RR = 1.19), polyp size ≥ 10 mm (RR = 2.43), polyp size > 10 mm (RR = 3.83), polyps located in the right semicolon (RR = 2.48) and endoscopic mucosal resection (RR = 2.99) were risk factors for DPPB.

**Conclusions:**

Male sex, hypertension, anticoagulation, polyp size, polyp size ≥ 10 mm, polyps located in the right semicolon, and endoscopic mucosal resection were the risk factors for DPPB. Based on our findings, we recommend that endoscopists should fully consider and implement effective intervention measures to minimize the risk of DPPB.

**Supplementary Information:**

The online version contains supplementary material available at 10.1186/s12876-024-03251-6.

## Background

Colonoscopy is very practical for screening and preventing colon cancer, and its importance is self-evident [1]. Endoscopic colorectal polypectomy has been proven to be an effective method to reduce the mortality of colorectal cancer [2]. With the continuous development of modern medicine and the continuous improvement of treatment technology, the safety of colorectal polypectomy has been improved to a certain extent, the number of outpatient or daytime operations has gradually increased, and the number of inpatient operations has gradually decreased. At present, the main methods of endoscopic resection of colorectal polyps include hot snare polypectomy (HSP), cold snare polypectomy (CSP), endoscopic mucosal resection (EMR), endoscopic submucosal dissection (ESD), hot biopsy (HB) and argon plasma coagulation (APC). Additionally, complications after colorectal polypectomy are still an important problem for clinicians.

Delayed postpolypectomy bleeding (DPPB) is one of the complications that can occur after colorectal polypectomy; it most often occurs after 24 h, with an incidence of 0.6%∼0.9% [3]. DPPB is difficult to detect via emergency endoscopy because the bleeding location is often hidden in the intestines covered by feces, thus increasing the burden on the doctors’ diagnosis and treatment work. Additionally, the problems caused by DPPB, such as patient discomfort, prolonged hospitalization, increased medical costs, and even increased patient mortality, can affect the harmony of the doctor-patient relationship.

There are different reports in the literature on factors related to DPPB. Some studies have shown that the incidence of DPPB is higher among patients receiving anticoagulant therapy [4–6]. Some studies also showed that the incidence of DPPB was positively correlated with the size of polyps removed [7–9]. A meta-analysis has concluded that cardiovascular disease, hypertension, polyps larger than 10 mm, and polyps located in the right colon are important risk factors for delayed bleeding [10].However, there remains a lack of clarity regarding the use of preventive measures on the wound surface during operation [11, 12], and whether such factors will affect the incidence of DPPB. Therefore, the risk factors of DPPB still need further verification. This study aims to update, comprehensively analyze, and explore the relevant risk factors for the occurrence of DPPB, to further optimize clinical response strategies and provide reference.

## Methods

Our search protocol was prospectively registered with PROSPERO (CRD 42,022,375,804). (Supplementary material 1)

### Literature retrieval

Three large Chinese databases were searched: CNKI database, Wan Fang database, and Wei Pu database. The databases were searched from inception to July 1, 2022. The key words were polypectomy or polypectomy or bleeding after polypectomy. Four large English databases were searched: Web of Science, PubMed, Cochrane Library, and Embase. The keywords were Polyp, Postoperative Hemorrhages, Risk Factors, and their free words. There were no language restrictions. The combination of subject words, keywords, and free words was used to search the databases and merge the search results. The search strategy is followed in Supplementary material 2.

### Inclusion criteria and exclusion criteria

The inclusion criteria were as follows: [1] prospective cohort study or retrospective case-control study; [2] an independent study with complete data and more than one control group with the same research purpose; [3] the study sample was adult patients (≥ 18 years old); and [4] the content of the study was the risk factors for delayed bleeding after colorectal polypectomy.

The exclusion criteria were as follows: [1] the research content did not involve or was not related to the risk factors for delayed bleeding after colorectal polypectomy; [2] duplicate publications repeatedly; [3] animal experiments, reviews, case reports, conference abstracts, dissertations; [4] studies without a control group; [5] the same researcher published similar documents; and [6] documents with incomplete data, unclear description, unavailable data, and limited research group.

### Literature screening and data extraction

All the studies were independently screened by two researchers (ZX, SL). The researchers screened the titles, abstracts, and full texts in accordance with the inclusion and exclusion criteria, and the data were extracted into an Excel spreadsheet. The extracted data included [1] general data, including first author’s name, year of publication, and type of study; [2] baseline data, including sample size, sex, and age; [3] patient factors; [4] polyp factors, including polyp number, polyp diameter, polyp shape, polyp location, and polyp pathological type; [5] operation factors; and [6] the same effect size and 95% confidence interval (CI) were obtained by multivariate regression analysis. Any disagreement was resolved through discussion, consultation with a third researcher (JX) if necessary, and discussion or arbitration.

### Quality assessment

Each study was independently evaluated by two researchers (ZX, SL). The Newcastle Ottawa scale was used to grade the included documents. The case-control study and cohort study were evaluated through three blocks and eight items, including the selection of study population, comparability, exposure evaluation or result evaluation. Any differences were resolved through discussion, and if necessary, a third researcher (JX) was consulted. The maximum score is 9 stars, and studies with ≥ 5 stars were included in the analysis.

### Statistical analysis

STATA 15.1 was used for data analysis, and *P* < 0.05 was considered statistically significant. The relative risk (RR) was used as the effect index for the secondary classification variable and the combined effect quantity, and the effect quantity was expressed by the 95% confidence interval (95% CI). *I*^*2*^ > 50% indicated substantial heterogeneity between studies, and in such cases, the random effects model was used to pool and analyze the data; when *I*^*2*^ < 50%, the fixed effects model was used to pool and analyze the data. When heterogeneity was observed, sensitivity analysis was used to further explore the source of heterogeneity. Publication bias was evaluated by a funnel chart.

## Results

### Literature retrieval process and results

**Fig. 1:**
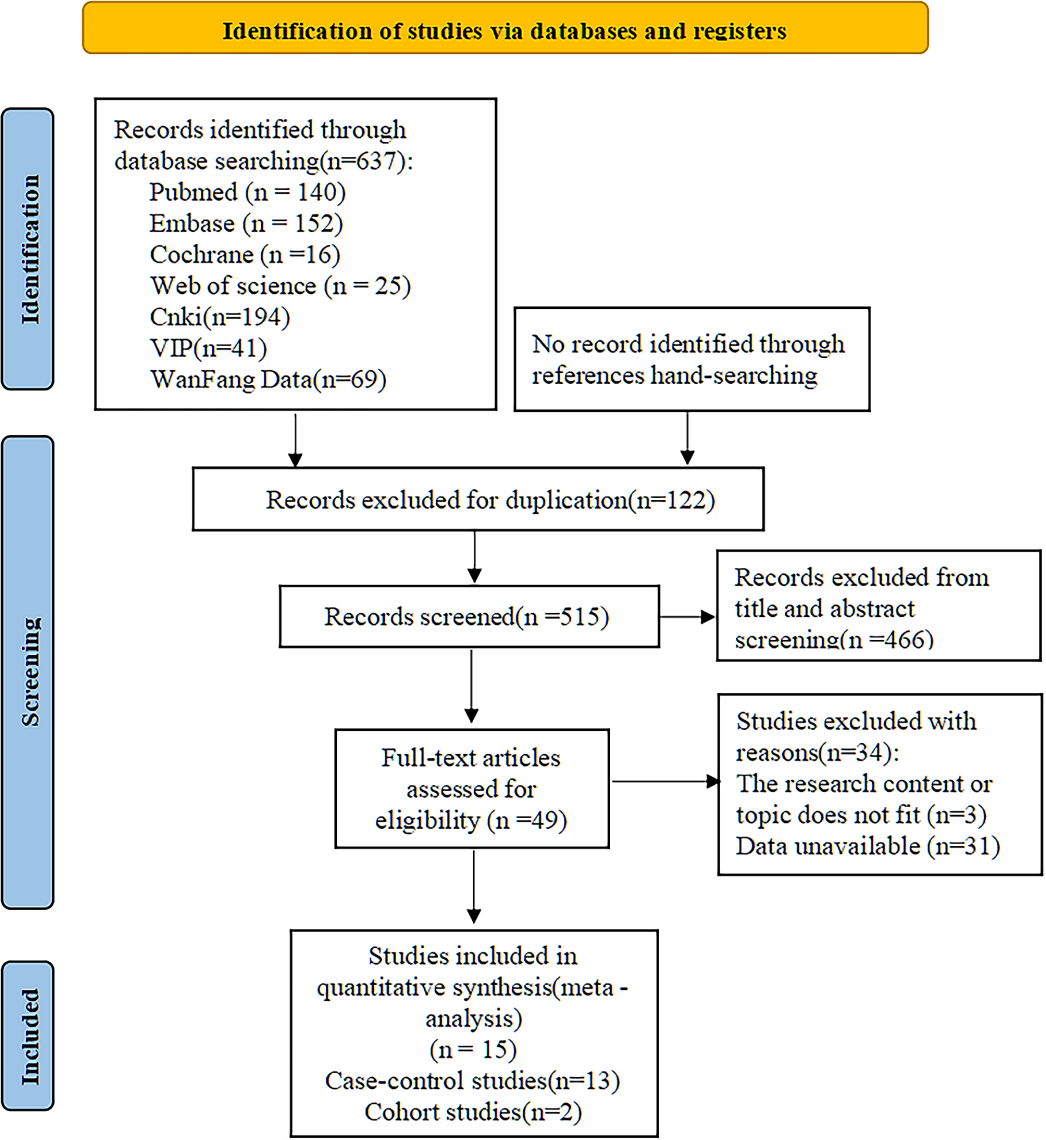
Flow chart of the literature screening process

A total of 637 relevant studies were retrieved. After screening, 15 articles were ultimately included, including 13 case-control studies, 2 cohort studies, 11 English studies and 4 Chinese studies. The literature screening process is shown in Fig. 1.

### Basic characteristics of the included studies

A total of 15 studies with 24,074 study subjects were included: 897 patients were in the bleeding group, and 23,177 patients were in the control group. Patients with DPPB were included in the study, while patients with no DPPB were in the control group. The basic characteristics of the included studies are shown in Table 1 and the excluded studies are shown in Supplementary material 3.

**Table 1:** Basic characteristics of the literature on risk factors for delayed postpolypectomy bleeding (DPPB).

Researcher	Year	Nation	Study Type	Sample size(*n*)	Gender		Age		NOS
					Male	Female	Bleeding Group	No Bleeding Group	
Yoshikazu Inagaki	2021	Japan	case-control	295	186	109	72.6 ± 8.3	68.6 ± 9.6	7
Xianyi Lin	2019	China	case-control	3962	2186	1776	51 ± 16	54 ± 10	6
Peipei Li	2019	China	case-control	287	199	88	56 ± 14	58 ± 12	6
Zhe Luo	2019	China	case-control	922	598	324	56.6 ± 12.3	58.8 ± 10.8	6
Changqin Liu	2019	China	case-control	709	468	241	62.71 ± 11.237	61.00 ± 9.376	7
Peng Cheng	2018	China	case-control	459	225	234	60.30 ± 10.66	58.16 ± 11.03	7
Soo Kyung Park	2018	Korea	prospective cohort study	3887	2661	1226	52.4 ± 12.3	55.8 ± 11.9	7
Bum Su Choung	2014	Korea	case-control	3788	2248	1540	60.21 ± 11.11	58.67 ± 11.40	7
Hee Seok Moon	2014	Korea	case-control	368	318	50	60.08 ± 13.36	60.62 ± 12.27	6
Qiang Zhang	2014	China	case-control	5600	3944	1656	47 ± 16	53 ± 14	7
Jeong Ho Kim	2013	Korea	case-control	210	155	55	58.0 ± 11.2	57.7 ± 11.2	8
Xianrui Wu	2013	America	prospective cohort study	120	62	58	69.9 ± 9.2	64.9 ± 12.2	6
K. Tim Buddingh	2011	Netherlands	case-control	156	73	80	66 ± 12	61 ± 12	8
M.S. Sawhney	2008	America	case-control	173	169	4	64.3 ± 16.7	65.4 ± 10.5	7
Hirotsugu Watabe	2006	Japan	case-control	3138	2578	560	61.4 ± 7.3	62.4 ± 10.1	6

NOS: Newcastle Ottawa scale

### Literature quality evaluation

A total of 6 studies scored 6 stars [6, 13–17], 7 studies scored 7 stars [3, 7, 8, 18–21], and 2 studies scored 8 stars [22, 23] (Table 1).

### Meta-analysis results

#### The incidence of DPPB

**Fig. 2:**
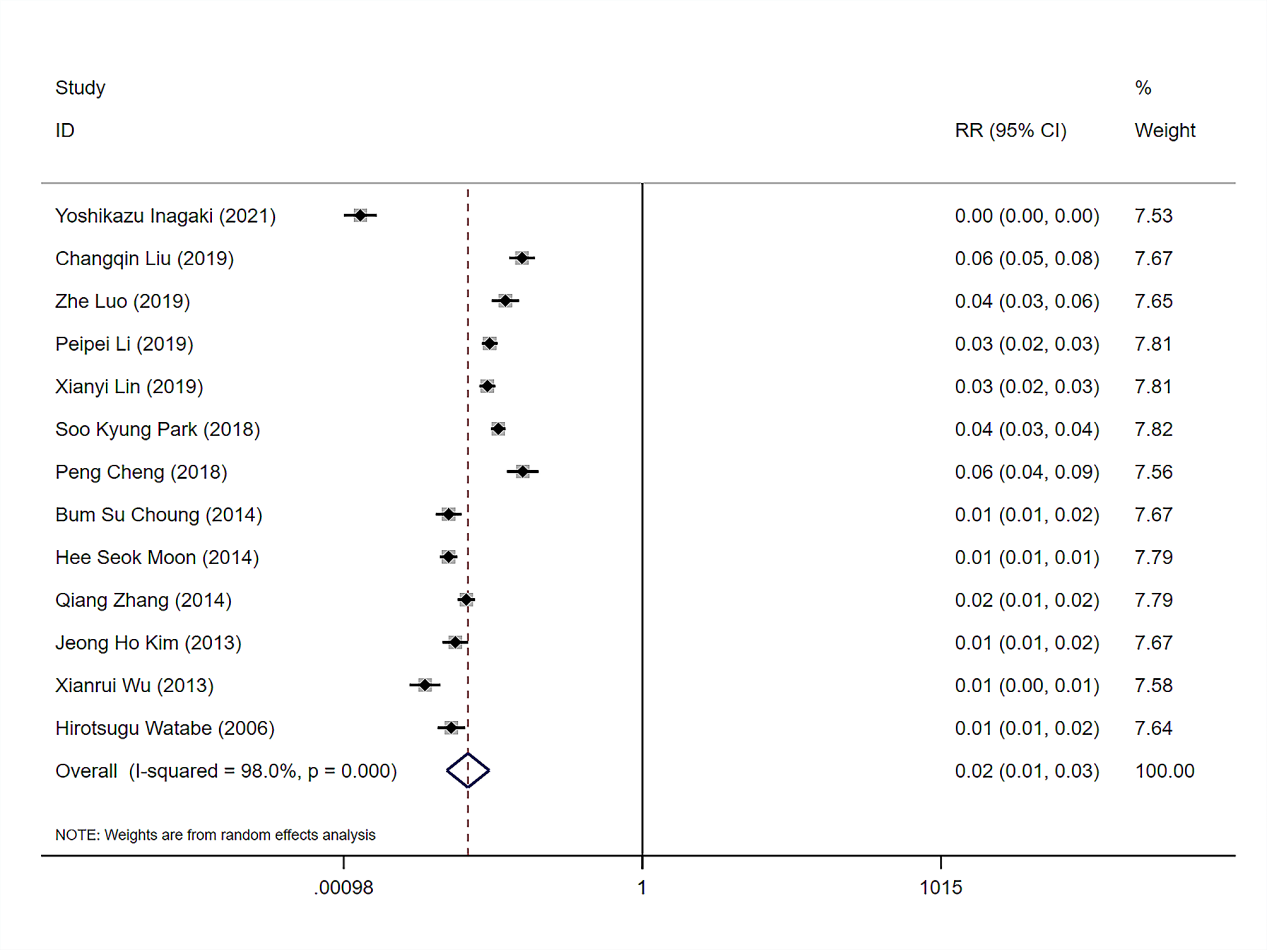
Forest plot of delayed postpolypectomy bleeding. RR: Relative Rate

Thirteen studies [3, 6–8, 13–20, 22] have examined the incidence of DPPB (i.e., the number of DPPB cases/total number of cases), with *I*^*2*^ = 98%. The random effects model was used for pooled analysis, and the incidence of DPPB was 0.02, 95% CI (0.01–0.03). Among the included studies, the highest incidence of DPPB was 0.06, 95% CI (0.05–0.08), while the lowest incidence of DPPB was 0.00, 95% CI (0.00–0.00) (Fig. 2).

#### Patient-related factors

The results showed that male sex (RR = 1.56, 95% CI: 1.34–1.81, *P* < 0.05), hypertension (RR = 1.27, 95% CI: 1.09–1.47, *P* < 0.05), cardiovascular disease (RR = 1.56, 95% CI: 1.23–1.97, *P* < 0.05), antithrombotic drugs (RR = 1.96, 95% CI: 1.24–3.09, *P* < 0.05), aspirin (RR = 1.50, 95% CI: 1.06–2.11, *P* < 0.05), and clopidogrel (RR = 1.89, 95% CI: 1.03–3.45, *P* < 0.05) (Fig.S1-6) were associated with increased risk of DPPB. Smoking, drinking, diabetes, cerebrovascular disease and warfarin were not related to the occurrence of DPPB (Table 2).

**Table 2:** Single-factor logistic regression effect value meta-analysis

Risk Factors		Study Number	Patient Number	Heterogeneity		RR 95% CI	*P*
				I² (%)	*P*		
Patient related factors							
Male sex		15	24,074	7.2	0.372	1.56(1.34–1.81)	0.001
Smoking		3	4830	82.4	0.003	1.36(0.50–3.66)	0.545
Drinking		2	3908	0.0	0.455	0.82(0.47–1.42)	0.477
Hypertension		11	14,885	22.8	0.226	1.27(1.09–1.47)	0.002
Diabetes		11	14,885	57.7	0.009	1.00(0.71–1.39)	0.983
Cardiovascular disease		9	10,539	0.0	0.549	1.56(1.23–1.97)	0.001
Cerebrovascular disease		4	1574	0.0	0.788	1.19(0.83–1.70)	0.336
Antithrombotic drugs		7	9257	77.0	0.000	1.96(1.24–3.09)	0.004
Aspirin		3	7923	46.2	0.156	1.50(1.06–2.11)	0.021
Clopidogrel		2	4082	0.0	0.363	1.89(1.03–3.45)	0.038
Warfarin		2	4082	81.7	0.020	2.70(0.57–12.68)	0.208
Polyp-related factors							
polyp number>3		2	7849	0.0	0.852	1.44(1.12–1.85)	0.005
polyp number ≥ 3		2	746	98.3	0.000	6.5(0.43–98.3)	0.176
Polyp size ≥ 10 mm		3	3061	2.3	0.359	3.57(2.58–4.95)	0.001
Pedunculated polyp		3	4457	0.0	0.727	4.32(2.97–6.30)	0.001
Polyp location	Left semicolon	8	18,161	68.5	0.002	0.84(0.59–1.20)	0.346
	Right semicolon	8	18,161	69.1	0.002	1.14(0.80–1.62)	0.482
Pathological type	Adenomas	7	14,199	80.6	0.000	1.70(0.89–3.23)	0.105
	Non-adenomas	2	754	72.3	0.058	0.44(0.08–2.51)	0.356
	Serrated polyps	3	9683	0.0	0.723	0.71(0.20–2.44)	0.584
	proliferative polyps	4	10,392	72.3	0.013	0.67(0.30–1.49)	0.327
	malignancies	5	13,445	0.0	0.933	2.66(1.49–4.75)	0.001
Operational related factors							
The way polyps removed	EMR	6	13,904	52.2	0.063	2.34(1.44–3.82)	0.001
	ESD	2	919	55.4	0.134	3.62(0.76–17.32)	0.107
	HSP	4	12,736	89.6	0.000	1.95(0.75–5.04)	0.168
	HB	4	12,985	0.0	0.974	0.28(0.17–0.46)	0.001
	APC	2	6309	99.6	0.000	3.60(0.00-52774.53)	0.794
Preventive wound management		2	3998	0.0	0.837	0.95(0.58–1.55)	0.834
Endoscopists	inexperienced	3	4792	87.9	0.000	1.55(0.42–5.69)	0.512
	experienced	3	4792	87.9	0.000	0.65(0.18–2.38)	0.514

APC: argon plasma coagulation; EMR: endoscopic mucosal resection; ESD: endoscopic submucosal dissection; HB: hot biopsy; HSP: hot snare polypectomy

**Fig. 3:**
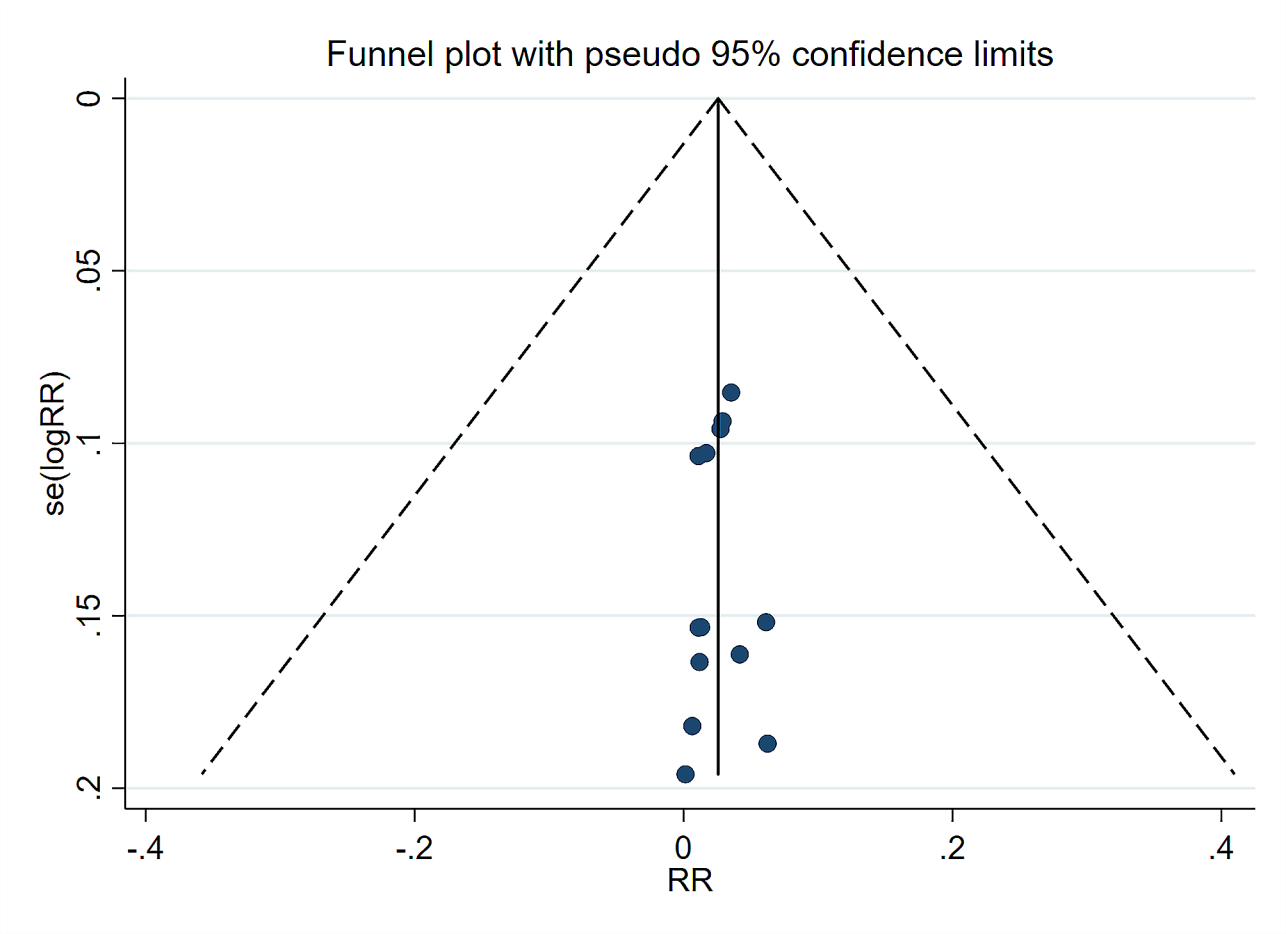
Funnel plot with 95% confidence limits of delayed postpolypectomy bleeding. RR: Relative Rate

#### Polyp-related factors

The results showed that the number of polyps > 3 (RR = 1.44, 95% CI: 1.12–1.85, *P* < 0.05), polyp size ≥ 10 mm (RR = 3.57, 95% CI: 2.58–4.95, *P* < 0.05), pedunculated polyps (RR = 4.32, 95% CI: 2.97–6.30, *P* < 0.05) and malignancies (RR = 2.66, 95% CI: 1.49–4.75, *P* < 0.05) (Fig. S7-10) were associated with an increased risk of DPPB. While polyp number ≥ 3, polyps located in left semicolon or the right semicolon, adenoma, non-adenoma, serrated polyp, and proliferative polyp were not associated with the incidence of DPPB (Table 2).

#### Operation-related factors

The results showed that EMR (RR = 2.34, 95% CI: 1.44–3.82, *P* < 0.05) (Fig.S11) was associated with an increased risk of DPPB, while HB (RR = 0.28, 95% CI: 0.17–0.46, *P* < 0.05) (Fig. S12) was associated with a decreased risk of DPPB. However, ESD, HSP, APC, preventive wound treatment, inexperienced endoscopists and experienced endoscopists were not associated with the occurrence of DPPB (Table 2).

#### Multivariate logistic regression effect value meta-analysis

The results showed male sex (RR = 1.64, 95% CI: 1.01–2.65, *P* < 0.05), hypertension (RR = 1.54, 95% CI: 1.15–2.07, *P* < 0.05), anticoagulation (RR = 4.04, 95% CI: 2.07–7.90, *P* < 0.05), polyp size (RR = 1.19, 95% CI: 1.10–1.30, *P* < 0.05), polyp size ≥ 10 mm (RR = 2.43, 95% CI: 1.80–3.29, *P* < 0.05), polyp size > 10 mm (RR = 3.83, 95% CI: 2.38–6.15, *P* < 0.05), polyp located in the right semicolon (RR = 2.48, 95% CI: 1.77–3.47, *P* < 0.05), and EMR (RR = 2.99, 95% CI: 1.06–8.45, *P* < 0.05) (Fig. S13-20) were associated with an increased risk of DPPB, while diabetes, aspirin, polyp number ≥ 3, pedunculated polyp, and HSP resection modes were not associated with the risk of DPPB (Table 3).

**Table 3:** Multivariate logistic regression effect value meta-analysis

Risk Factors		Study Number	Patient Number	Heterogeneity		RR 95% CI	*P*
				I² (%)	*P*		
		Patient related factors					
Male sex		4	4843	29.5	0.235	1.64(1.01–2.65)	0.045
Hypertension		7	15,526	31.8	0.186	1.54(1.15–2.07)	0.004
Diabetes		3	7198	33.3	0.198	1.57(0.73–3.37)	0.243
Anticoagulation		2	3961	0.0	0.373	4.04(2.07–7.90)	0.001
Aspirin		3	8022	45.0	0.162	1.37(0.74–2.54)	0.317
		Polyp-related factors					
polyp number ≥ 3		2	746	96.4	0.001	13.04(0.42-407.67)	0.144
Polyp size		4	4659	72.3	0.013	1.19(1.10–1.30)	0.001
Polyp size	≥ 10 mm	5	7981	3.7	0.385	2.43(1.80–3.29)	0.001
	>10 mm	2	9388	30.4	0.231	3.83(2.38–6.15)	0.001
Pedunculated polyp		4	4630	81.0	0.001	1.92(0.80–4.63)	0.147
Right semicolon		5	8409	0.0	0.571	2.48(1.77–3.47)	0.001
		Operational related factors					
Resection method	EMR	4	9906	58.0	0.067	2.99(1.06–8.45)	0.039
	HSP	2	8738	0.0	0.539	1.78(0.80–3.97)	0.157

EMR: endoscopic mucosal resection; HSP: hot snare polypectomy

#### Publication bias evaluation

Visual inspection of the funnel plot was conducted to check for publication bias. Regarding the incidence of DPPB and the outcome with the largest number of included studies (13 articles), the funnel plot was observed to be symmetrical, indicating that there was no significant publication bias in the DPPB bleeding rate, as shown in Fig. 3.

#### Sensitivity analysis

In this study, when the amount of heterogeneity for a factor was high (*I*^*2*^ > 50%), and the difference was statistically significant, sensitivity analyses were performed. Regarding multivariate logistic regression meta-analysis, EMR, sensitivity analysis (Fig. S20) revealed that excluding the study by Changqin Liu led to results that were outside of the 95% CI as well as a lower level of heterogeneity (*I*^*2*^ = 0.0%, *P* = 0.758) (Fig. S21). Therefore, this study may be a significant source of heterogeneity in the multivariate analysis of EMR. Regarding the polyp size and pedunculated polyp, sensitivity analysis revealed that the results remained within the 95% CI, and thus, the findings were stable. Due to the small number of included studies (2 articles), sensitivity analysis could not be performed to examine polyp number ≥ 3. Regarding single-factor logistic regression effect value meta-analysis, sensitivity analysis also revealed that the effects of smoking, diabetes, antithrombotic drugs, polyp located in left semicolon, right semicolon, adenoma, proliferative polyps, EMR, HSP, experienced and inexperienced endoscopists on the incidence of DPPB were stable. Due to the small number of included studies (2 articles), sensitivity analysis could not be performed to examine warfarin, polyp number ≥ 3, non-adenoma, ESD, and APC.

## Discussion

As one of the complications that can occur after endoscopic resection of colorectal polyps, DPPB may cause hemorrhagic shock and increase the risk of mortality if it is not treated in a timely manner [24]. Moreover, most DPPB patients need to undergo colonoscopy again, thereby increasing patients’ pain and economic losses. Therefore, DPPB is an important problem for endoscopists, but its etiology and mechanism remain unclear. Domestic and foreign studies have reported that the occurrence of DPPB is related to a variety of factors. The current meta-analysis included both univariate logistic regression and multivariate logistic regression, and the results revealed that male sex, hypertension, Anticoagulation, polyp size, polyp size ≥ 10 mm, polyp located in the right semicolon, and EMR were risk factors for DPPB. The results of multivariate logistic regression meta-analysis showed a significant correlation, while the results of univariate logistic regression meta-analysis revealed polyps located in the right half colon were not associated with the risk of DPPB, which might be attributed to the correlation between it and confounding factors.

Regarding patient-related factors, this study found that male patients were more likely to develop DPPB. The reason for this association may be related to women are more likely to follow behavioral instructions [25]. Vascular endothelial cell dysfunction in patients with hypertension can seriously affect the systolic blood pressure, the diastolic function of blood vessels, and vascular sclerosis; furthermore, this dysfunction can lead to decreased blood elasticity and significantly decreased contractility of blood vessels at the broken end [26]. In addition, the effects of atherosclerosis and the elasticity of blood vessels are further weakened. Furthermore, blood pressure fluctuates greatly, and hemodynamics are unstable, which easily causes blood vessel rupture and bleeding at the cutting site [27]. This study found that oral antithrombotic drugs could increase the incidence of DPPB, which was consistent with the conclusions of Bum Su Choung et al. [19, 21] and Xianyi Lin et al. [13, 18, 21]. For the timing of antithrombotic drugs use, one research was screened for discontinuation of anticoagulants for 5 days [19], and the other research restricted the use of anticoagulants to heparin or warfarin within 1 week after polypectomy [21]. This may lead to heterogeneity and affect the results. The analysis of NOAC was not mentioned in this study because of the lack of exact data on specific NOAC in the included literature.

Previous studies have reported that polyp size was one of the important factors affecting the incidence of DPPB [13, 15, 21, 23]. Our meta-analysis also found that a larger polyp size was associated with a greater risk of DPPB, especially when the size of the removed polyp was greater than or equal to 10 mm. This association may be due to larger polyps’ size being associated with more nourishing blood vessels, larger wound caused by resection, a greater extent of damage to blood vessels, and increased difficulty with repairing the blood vessels. It remains unclear whether polyp location affects the risk of DPPB after colorectal polypectomy. Que et al. reported that the position of polyps in the right half colon or rectum was a risk factor for delayed postoperative bleeding [12]. Eleftheriadis D et al. found that delayed bleeding was more likely to occur after right half colon polypectomy [11]. However, Inagaki Y et al. found that DPPB was more likely to occur when the lesions were in the rectum [7].The results of this study showed that the removal of polyps located in the right colon increased the risk of DPPB. Previous studies [15, 18, 19, 22, 23] suggest that this association may be related to the histological variation in colon location (thinner submucosa) and the different manipulation techniques required at this site [19].

Regarding operational related factors, we found that EMR resection were more likely to develop DPPB. The reason may be attributed to the fact that EMR are primarily targeted towards polyps with a larger diameter, as these tend to have a higher vascularity. Inadequate handling of blood vessels during the procedure can potentially lead to delayed bleeding [3]. Argon plasma coagulation (APC) was used to remove smaller polyps (Diameter less than 5 mm) [20],while there is no significant association between it and DPPB was found in this study.

As for the prophylactic use of preventive endoscopic clip closure, only one of the included literatures met the criteria [19], so the relationship between preventive endoscopic clip closure and the risk of DPPB could not be analyzed, and further research is needed.

Some factors were significantly correlated with an increased risk of DPPB in the single-factor logistic regression meta-analysis but not in the multivariate logistic regression meta-analysis, including cardiovascular disease, aspirin, clopidogrel, polyp number > 3, pedunculated polyps, pathological type of malignancies, and treatment method of HB. The reason for this inconsistency may be the existence of a false correlation or indirect correlation between these factors and the occurrence of DPPB. Once other factors are added, the false correlation disappears, indicating that they may not actually be risk factors for the occurrence of DPPB. Furthermore, this inconsistency may indicate potential publication bias.

This study has some limitations. First, this meta-analysis was based on 13 case-control studies and 2 prospective cohort studies, therefore confounding is possible. Second, we found significant heterogeneity among the analyses of polyp number, polyp size, and polyp shape, which may be related to differences in study populations and study designs. Third, due to the limited number of included studies, the relationship between cold snare, preventive endoscopic clip closure and DPPB occurrence could not be analyzed.

## Conclusion

In conclusion, colon colorectal polypectomy is an effective method for the prevention and treatment of colorectal cancer. However, regardless of how experienced the endoscopists are, there is always a risk of delayed postoperative bleeding. There are many risk factors for DPPB, so endoscopists should fully consider and implement effective intervention measures. Considerable attention should be devoted to patients with the following risk factors: male sex, hypertension, Anticoagulation, polyp size, polyp size ≥ 10 mm, polyp located in the right semicolon and EMR. During the operation, precise hemostasis should be performed, and drug therapy and active and rigorous follow-up should be used after surgery to form a multilink precise preventive intervention system. As it is difficult to eliminate the influence of the confounding factors examined herein, the conclusions of this study need to be further confirmed by more clinical controlled studies.

### Electronic supplementary material

Below is the link to the electronic supplementary material.

Supplementary Material 1

Supplementary Material 2

Supplementary Material 3

Supplementary Material 4

## Data Availability

The datasets used and/or analyzed during the current study are available from the corresponding author on reasonable request.

## Abbreviations

DPPB: Delayed postpolypectomy bleeding
RR: Relative risk
CI: Confidence interval
HSP: Hot snare polypectomy
CSP: Cold snare polypectomy
EMR: Endoscopic mucosal resection
ESD: Endoscopic submucosal dissection
HB: Hot biopsy
APC: Argon plasma coagulation
