# Supplementary material for: Risk factors for delayed colorectal postpolypectomy bleeding: a meta-analysis
Source: BMC Gastroenterol. 2024 May 14;24:162. doi: 10.1186/s12876-024-03251-6 (PMC11092041; doi:10.1186/s12876-024-03251-6)
Supplement: Supplementary file 2 — Supplementary Material 2 [file 12876_2024_3251_MOESM2_ESM.docx]

**Supplementary material 3** Excluded studies.

| **Title** | **The first author** | **Reasons for exclusion** |
| --- | --- | --- |
| Blood supply of colorectal polyps correlates with risk of bleeding after colonoscopic polypectomy | Dobrowolski, S. | The research content or topic does not fit |
| Factors associated with delayed bleeding after resection of large nonpedunculated colorectal polyps | Elliott, T. R. | The research content or topic does not fit |
| Incidence and predictors of postpolypectomy bleeding in colorectal polyps larger than 10 mm. | Gimeno-García Antonio Z | The research content or topic does not fit |
| Risk factors for post-polypectomy bleeding; a retrospective case-control study of a high-volume colonoscopy center | Eleftheriadis, D. | Data unavailable |
| Risk factors for delayed bleeding after endoscopic mucosal resection of colonic polyps | Yongchao Li | Data unavailable |
| Risk factors of delayed colorectal bleeding following endoscopic polypectomy | Yangming Que | Data unavailable |
| Risk factors of delayed bleeding after colorectal polypectomy | Jiaqi Wang | Data unavailable |
| Analysis of the risk factors for delayed colorectal post-polypectomy bleeding | Yuanran Chen | Data unavailable |
| Analysis of risk factors and risk line graph prediction model for gastrointestinal bleeding after endoscopic resection of colon polyps | Mingjun Gao | Data unavailable |
| Risk factors of delayed postpolypectomy bleeding | Liyun Huang | Data unavailable |
| Risk factors for delayed bleeding after endoscopic polypectomy of colorectal polyps | Miao Liu | Data unavailable |
| An analysis on relevant factors of tardive bleeding post resection under endoscope for intestinal polyps | Linchen Tang | Data unavailable |
| Clinical Efficacy of Endoscopic Mucosal Resection in Treatment of Patients with Colorectal Polyps and Analysis of Related Risk Factors for Delayed Hemorrhage after Surgery | Yaling Wan | Data unavailable |
| Analysis of risk factors for delayed bleeding after endoscopic colorectal polypectomy | Zhihua Zhang | Data unavailable |
| Study on related factors of hemorrhage after colorectal polypectomy | Zongguang Fu | Data unavailable |
| A study of risk factors for delayed bleeding after EMR for colon polyps | Yifeng Jin | Data unavailable |
| Analysis of risk factors associated with the occurrence of bleeding after endoscopic resection treatment of intestinal polyps | Bingjie Leng | Data unavailable |
| Analysis of factors predisposing to the occurrence of bleeding after electrodesiccation of colon polyps | Chengyi Liu | NData unavailable |
| Analysis of Influencing Factors of Delayed Bleeding after Endoscopic Col-orectal Polypectomy | Wenqin Liu | Data unavailable |
| Influencing factors of delayed bleeding after endoscopic polypectomy in patients with colonic polyps | Aiwen Ma | Data unavailable |
| An analysis of risk factors leading to delayed postoperative traumatic bleeding in patients undergoing endoscopic colorectal polypectomy | Meiqin Liu | Data unavailable |
| An analysis of risk factors for delayed hemorrhage following endoscopic colorectal polypectomy | Xiaocui Ru | Data unavailable |
| Clinical study of risk factors and treatment strategies for bleeding after colonic polypectomy | Ganhua Guo | Data unavailable |
| A study of risk factors for delayed bleeding after endoscopic resection of colorectal polyps | Hu Tang | Data unavailable |
| Inducing factors of delayed bleeding after endoscopic mucosal resection for colorectal polyp | Xingsheng Wang | Data unavailable |
| Analysis of factors associated with delayed bleeding after endoscopic resection of colorectal polyps | Chunming Yang | Data unavailable |
| Risk factors analysis of bleeding after endoscopic polypectomy of colon | Jinlu Yang | Data unavailable |
| Risk factors of delayed hemorrhage after endoscopic mucosal resection for colorectal polyp | Meihua Yang | Data unavailable |
| Analysis of factors associated with delayed bleeding after colonoscopic colorectal polypectomy | Jianhai Zhang | Data unavailable |
| Risk factors of delayed bleeding after endoscopic resection of colorectal polyps | Kaijun Zhang | Data unavailable |
| Analysis of risk factors for bleeding after endoscopic resection of colorectal polyps | Lijuan Zhang | Data unavailable |
| Risk factors for delayed bleeding after colorectal polypectomy | Huapin Xie | Data unavailable |
| Analysis of delayed bleeding after polypectomy with colonoscopy | Yinbin Zhou | Data unavailable |
| Analysis of risk factors associated with bleeding after endoscopic treatment of intestinal polyps | Jian Zou | Data unavailable |
