# Supplementary material for: Risk factors for delayed colorectal postpolypectomy bleeding: a meta-analysis
Source: BMC Gastroenterol. 2024 May 14;24:162. doi: 10.1186/s12876-024-03251-6 (PMC11092041; doi:10.1186/s12876-024-03251-6)
Supplement: Supplementary file 3 — Supplementary Material 3 [file 12876_2024_3251_MOESM3_ESM.doc]

**Supplementary Digital Content**

**Part 1: Single factor logistic regression effect value meta-analysis forest plots**


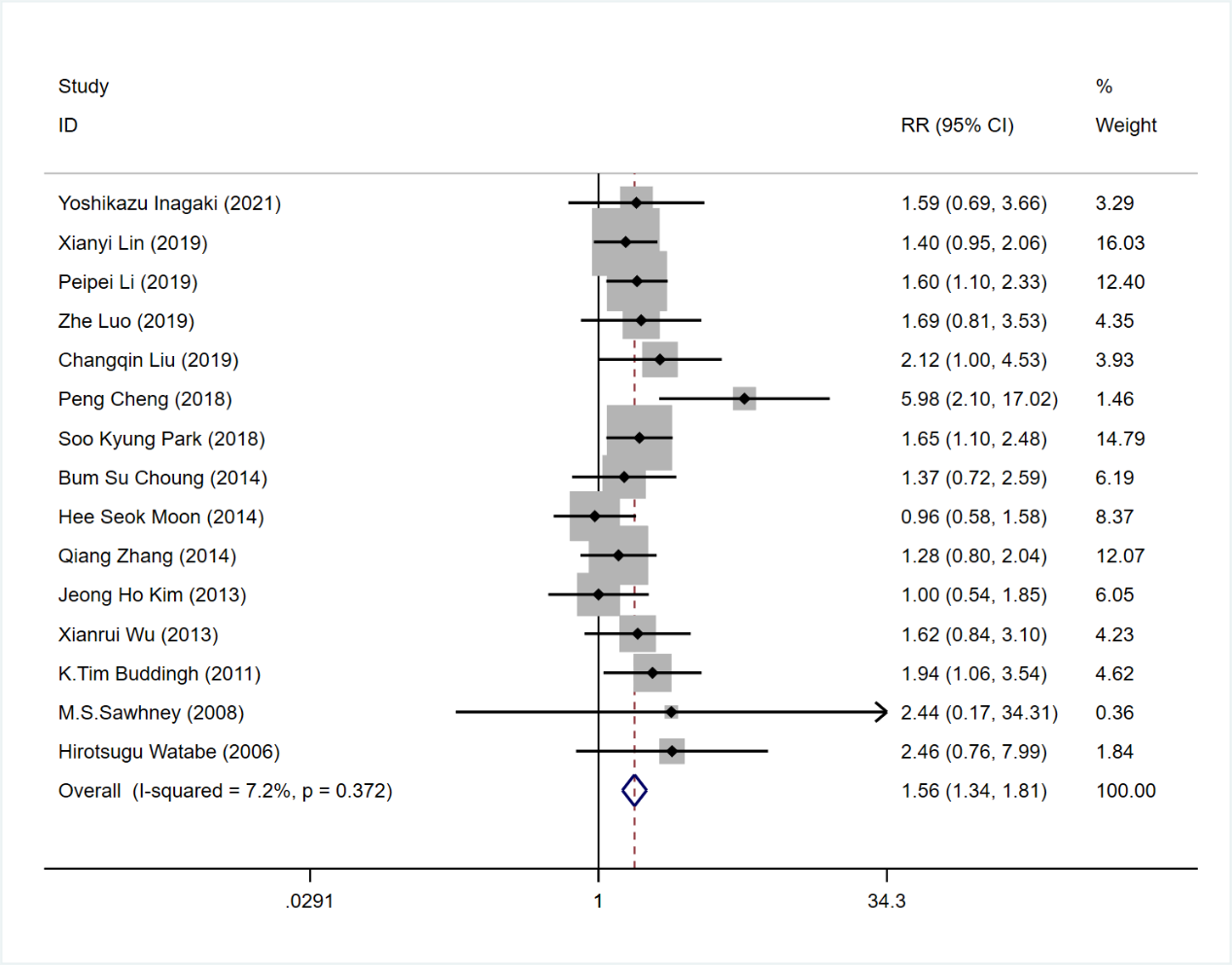


**Fig. S1.** Forest plot of male sex.


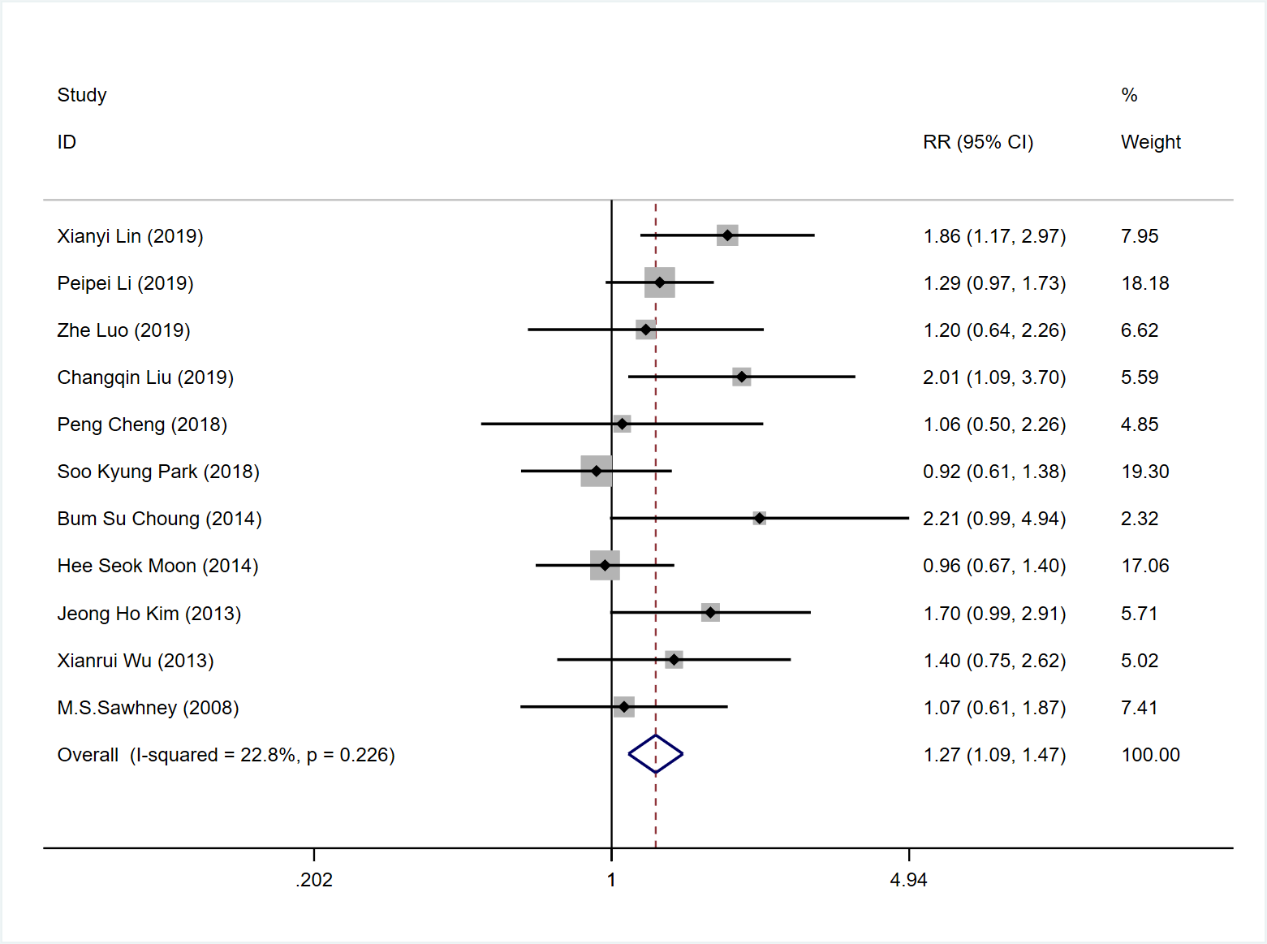


**Fig. S2.** Forest plot of hypertension.


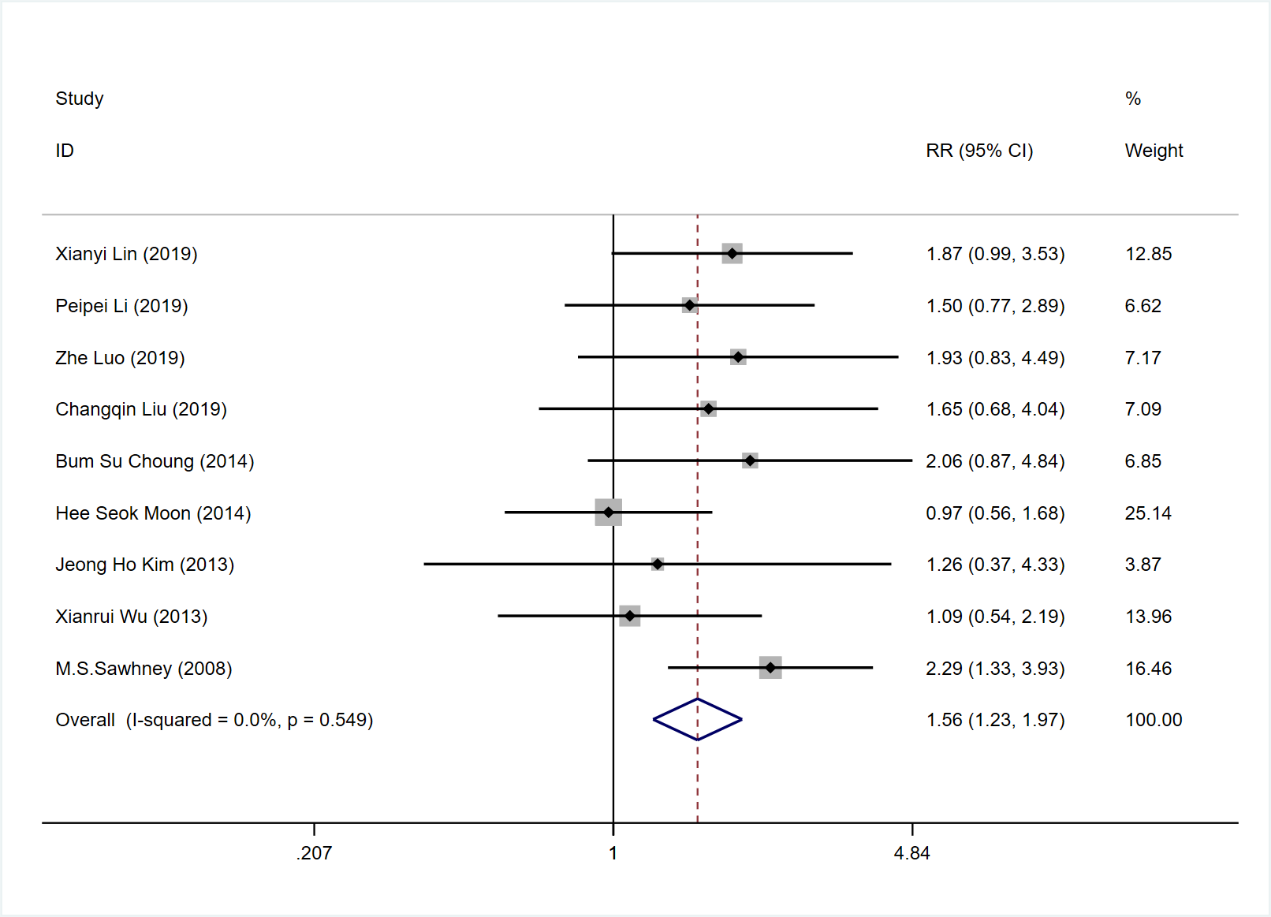


**Fig.** **S3.** Forest plot of cardiovascular disease.


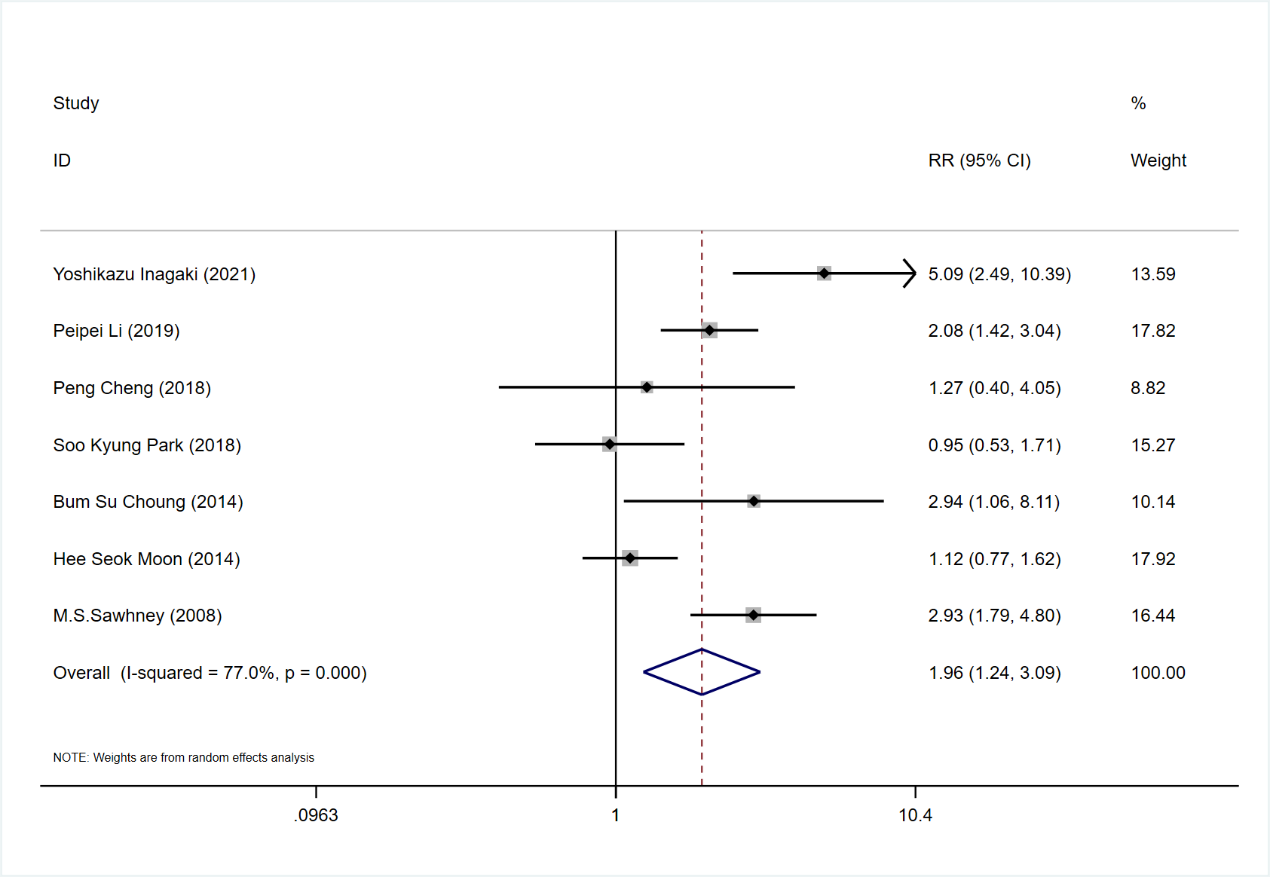


**Fig. S4.** Forest plot of antithrombotic drugs.


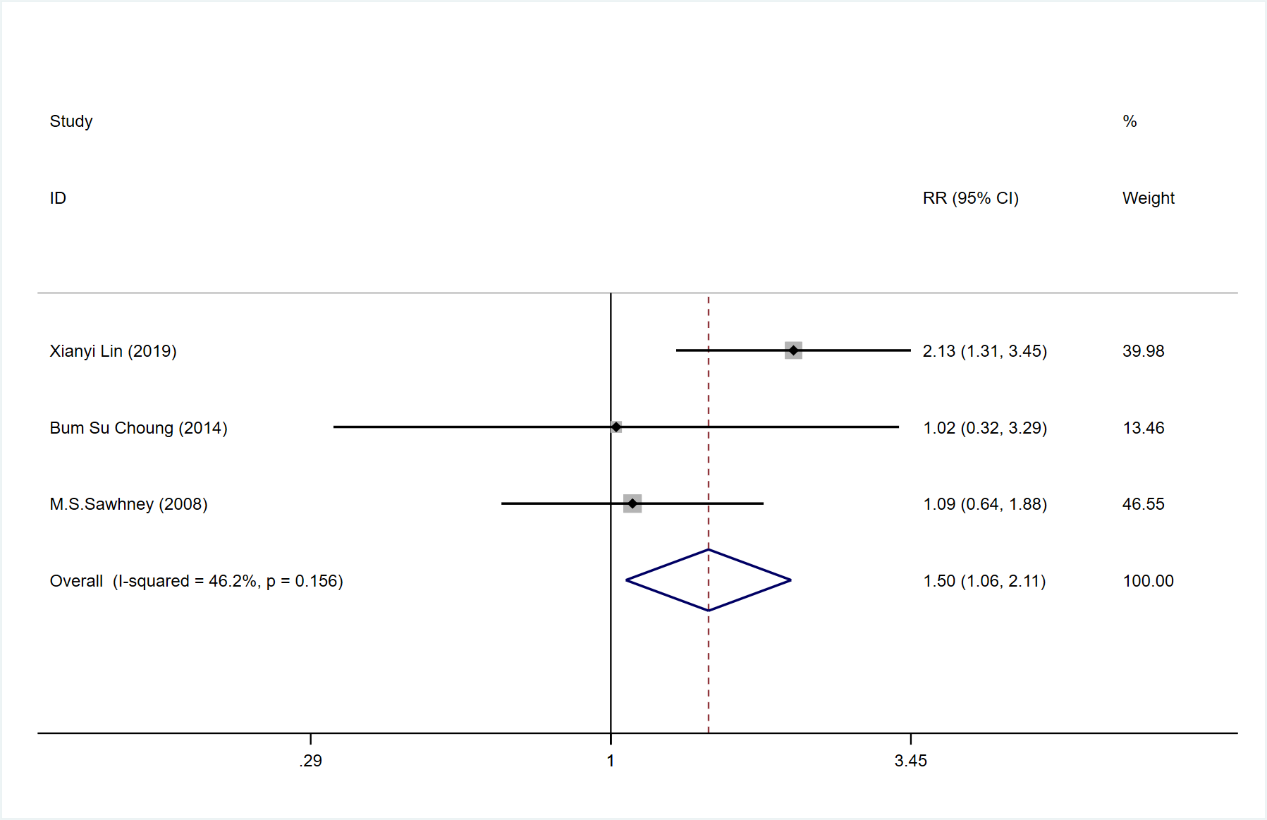


**Fig. S5.** Forest plot of aspirin.


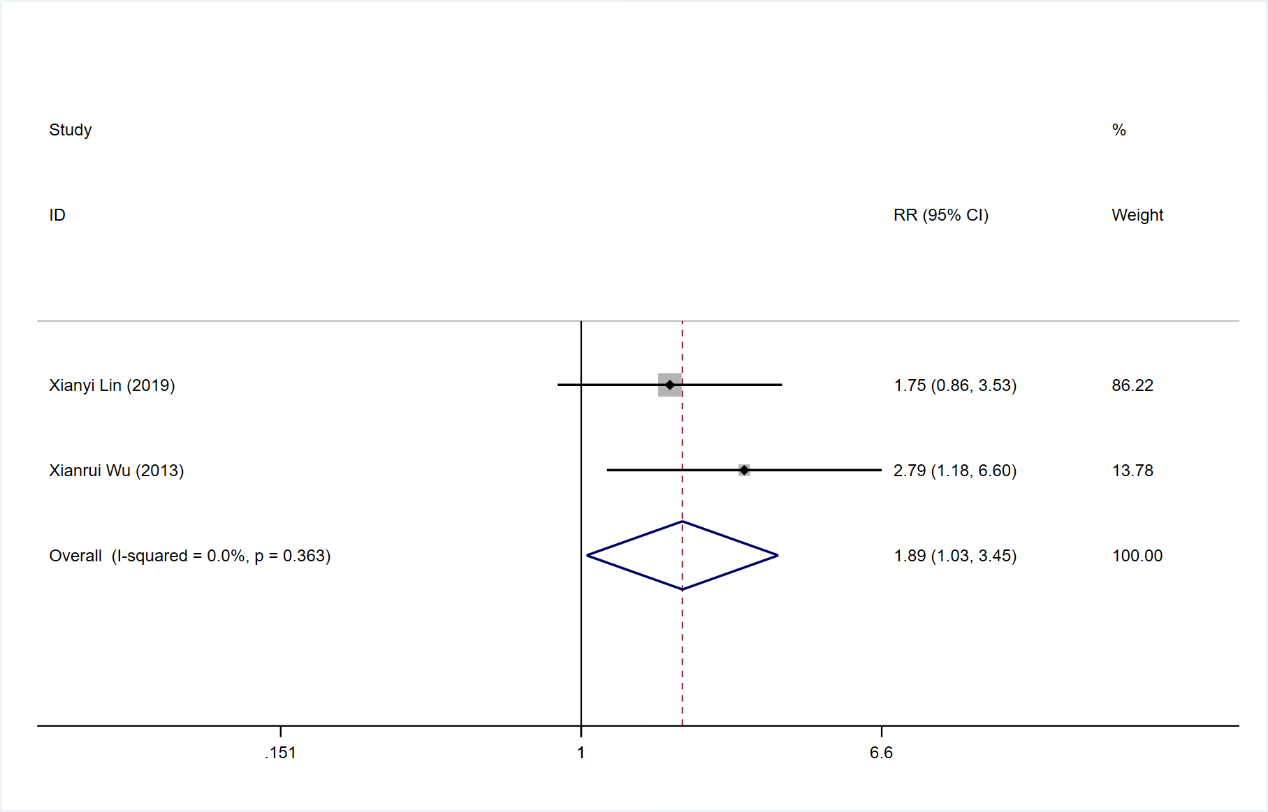


**Fig. S6.** Forest plot of clopidogrel.


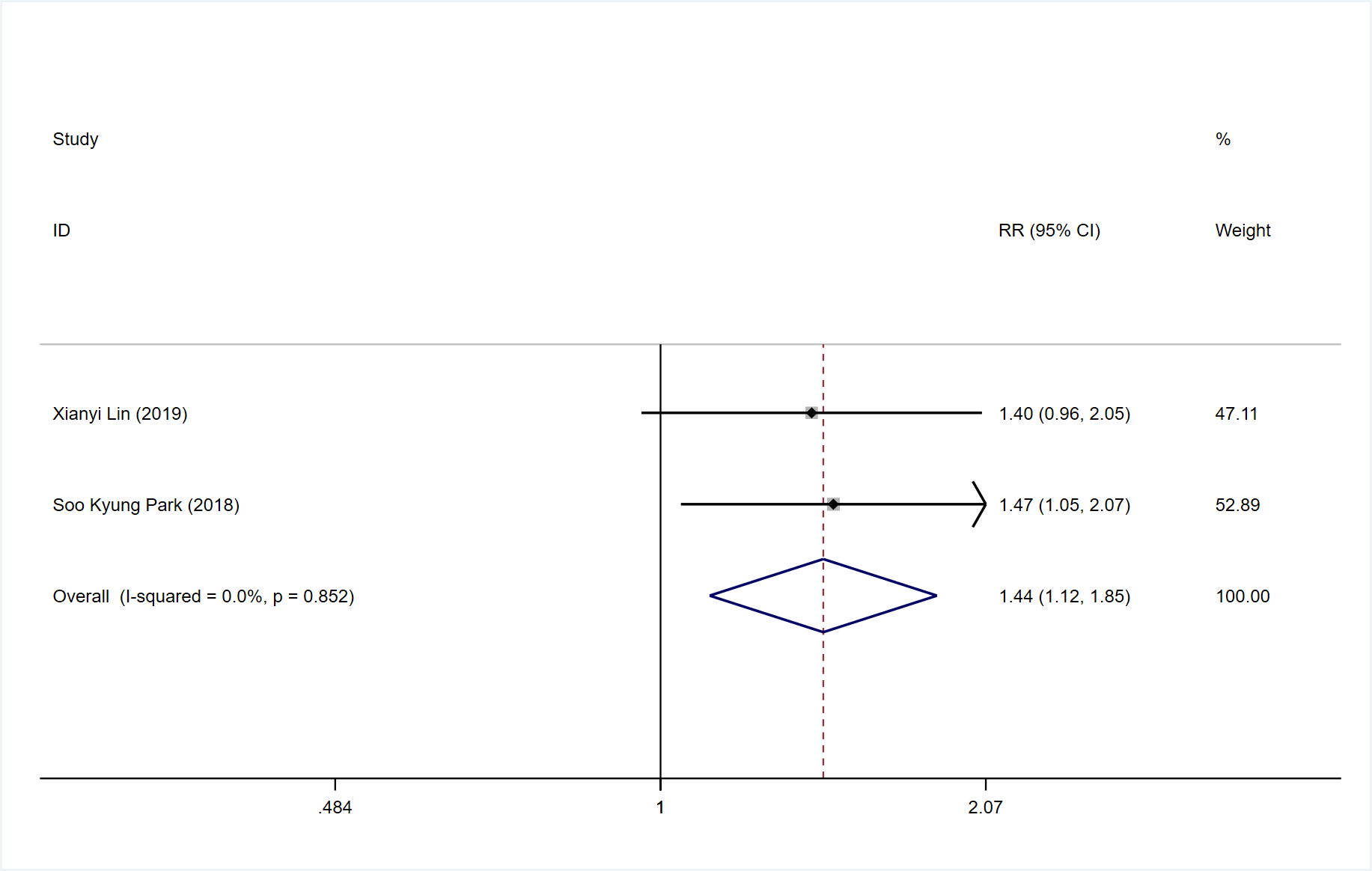


**Fig. S7.** Forest plot of polyp number > 3.


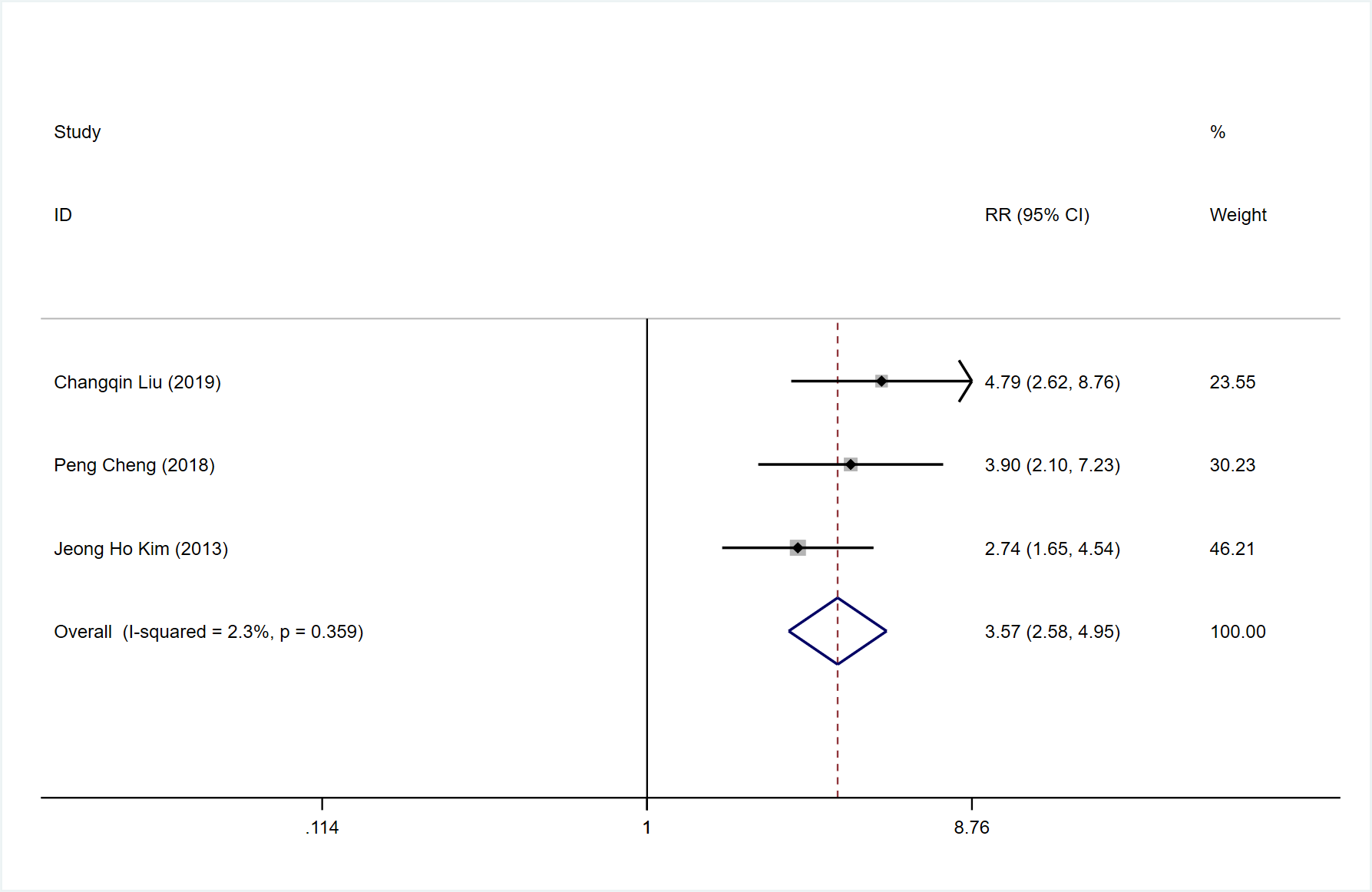


**Fig. S8.** Forest plot of polyp size ≥ 10 mm.

**
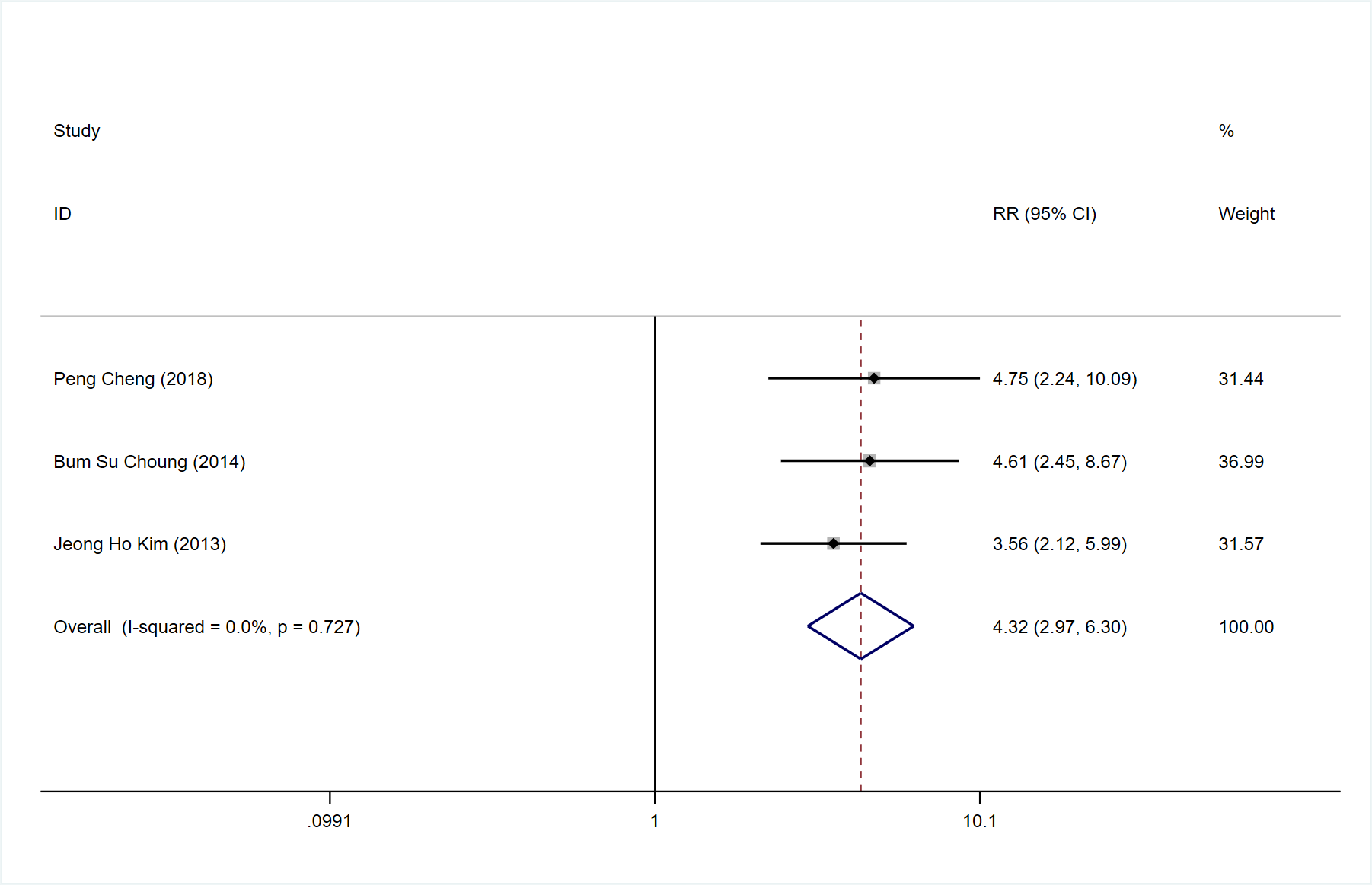
**

**Fig. S9.** Forest plot of pedunculated polyps.

**
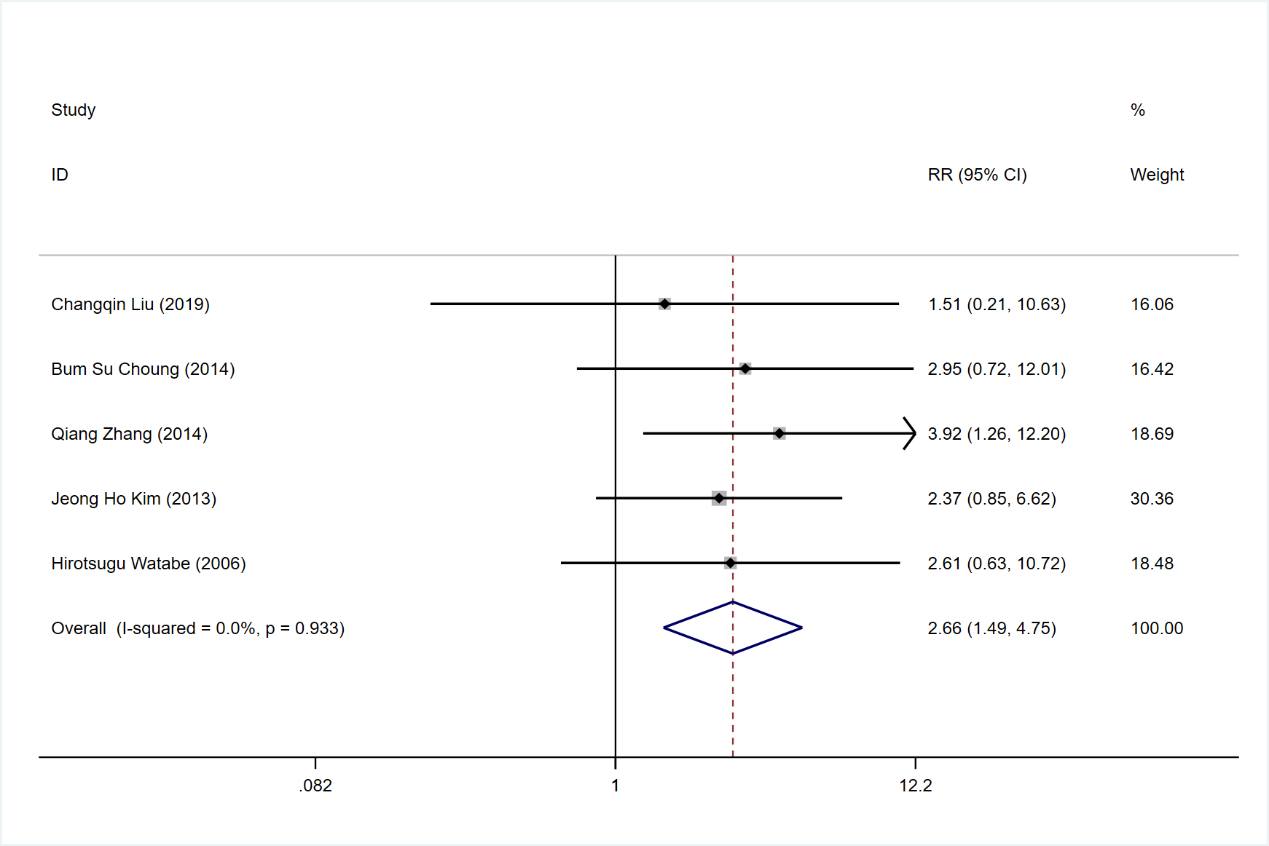
**

**Fig. S10.** Forest plot of malignancies.


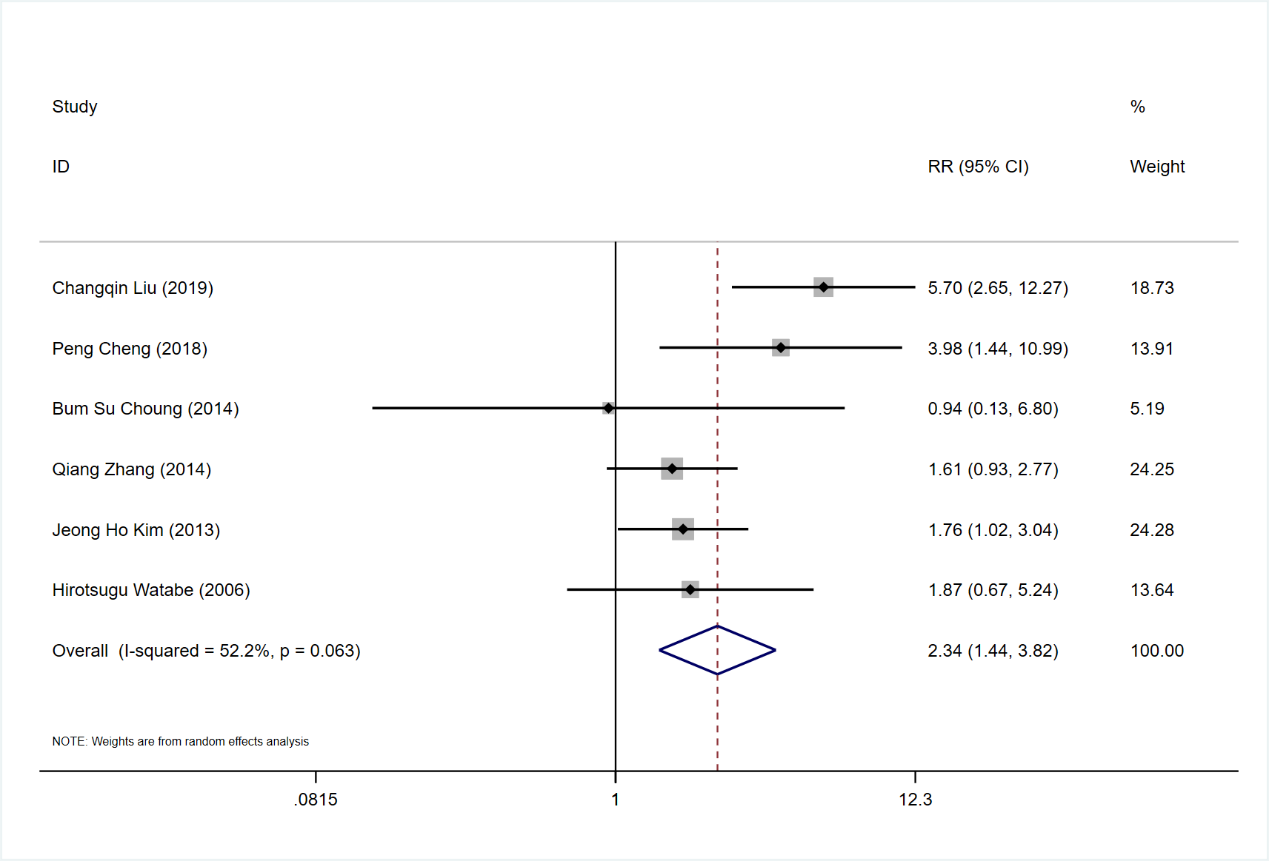


**Fig. S11.** Forest plot of endoscopic mucosal resection.

**
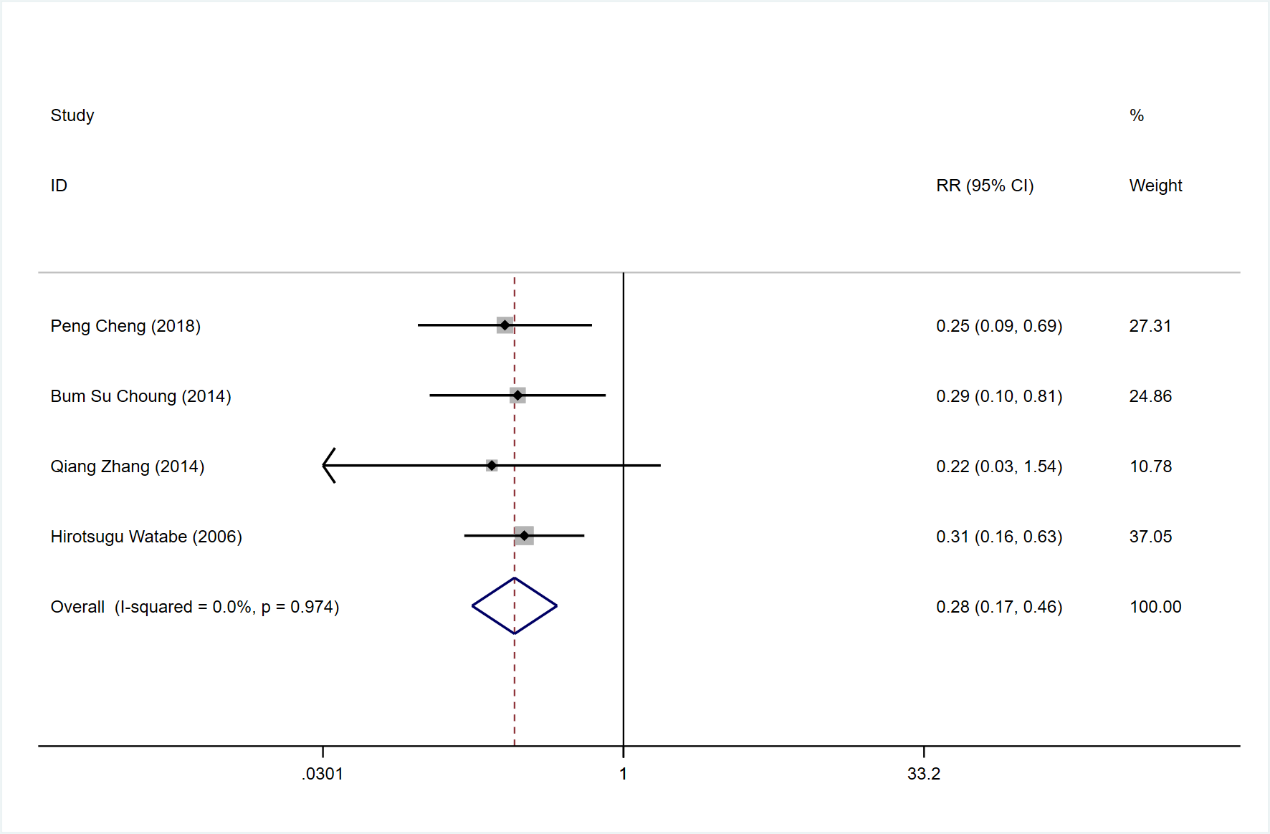
**

**Fig. S12.** Forest plot of hot biopsy.

**Part 2: Multivariate logistic regression** **effect value meta-analysis forest plots**

**
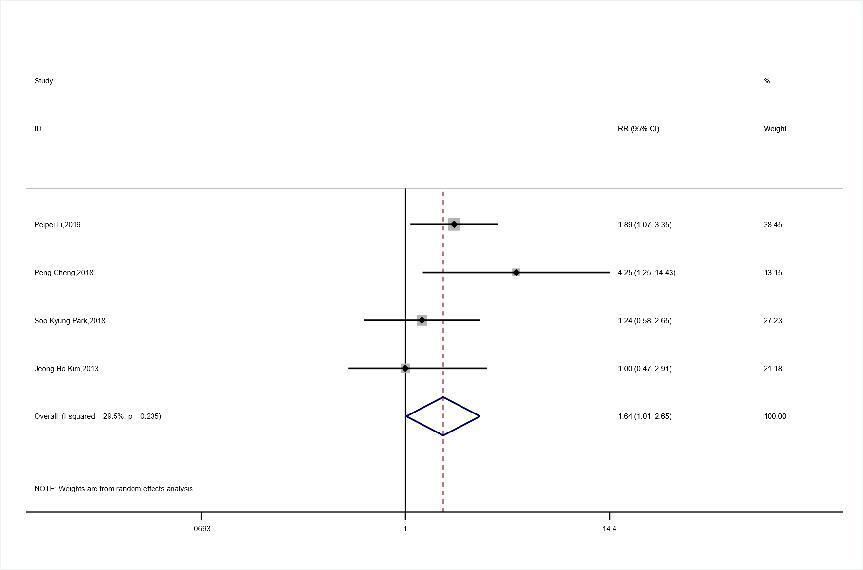
**

**Fig. S13.** Forest plot of male sex**.**

**
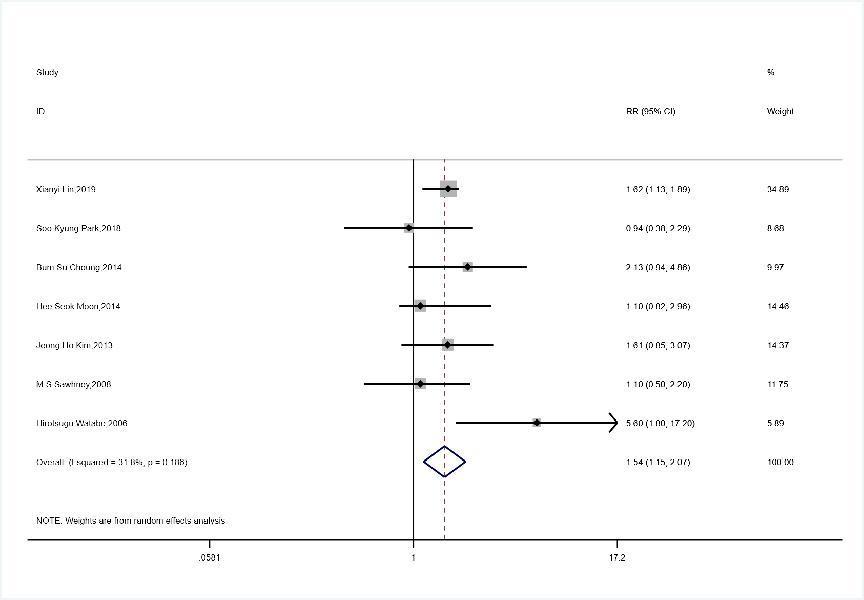
**

**Fig. S14.** Forest plot of hypertension.

**
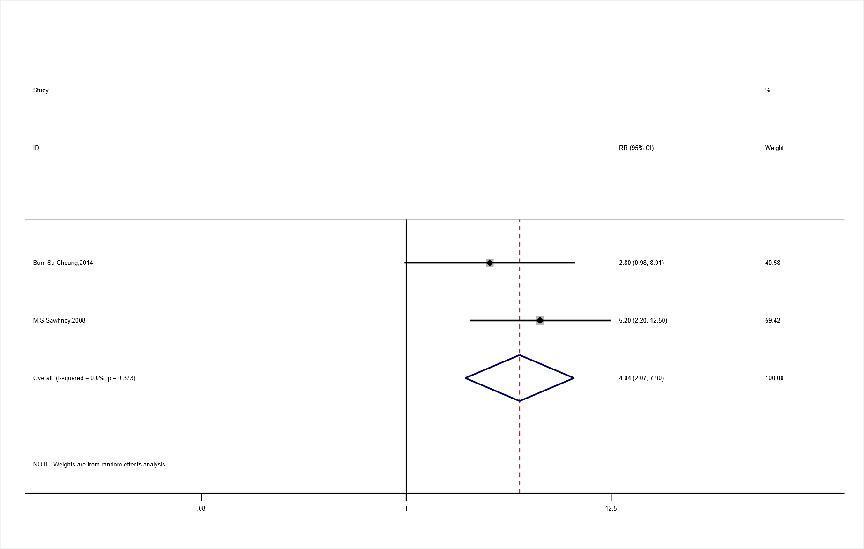
**

**Fig. S15.** Forest plot of anticoagulation.


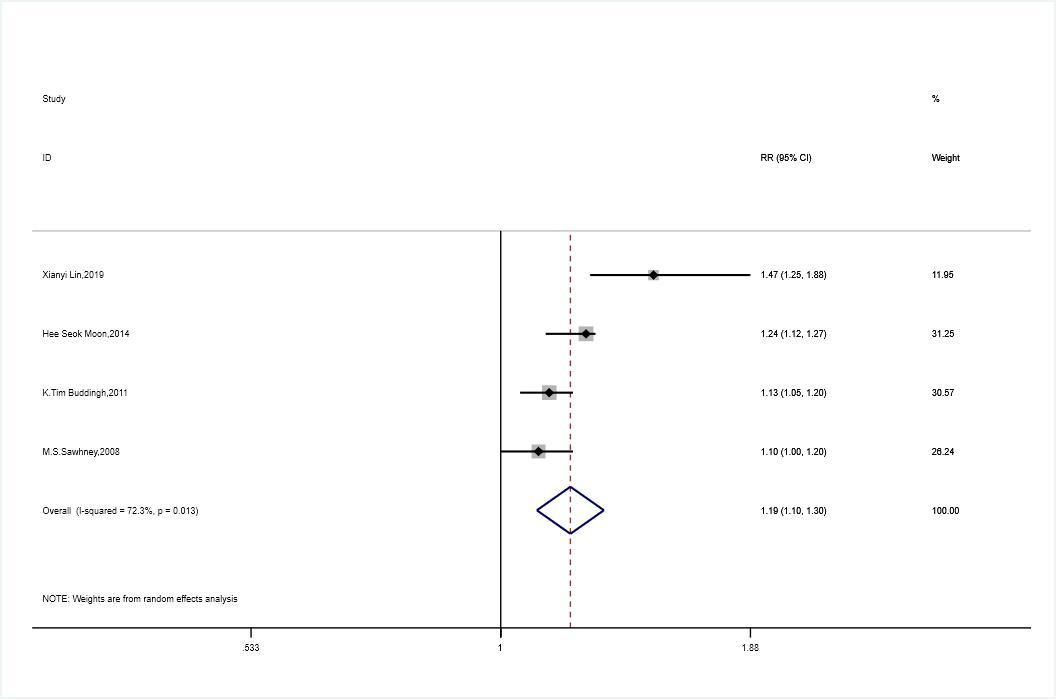


**Fig. S16.** Forest plot of polyp size.


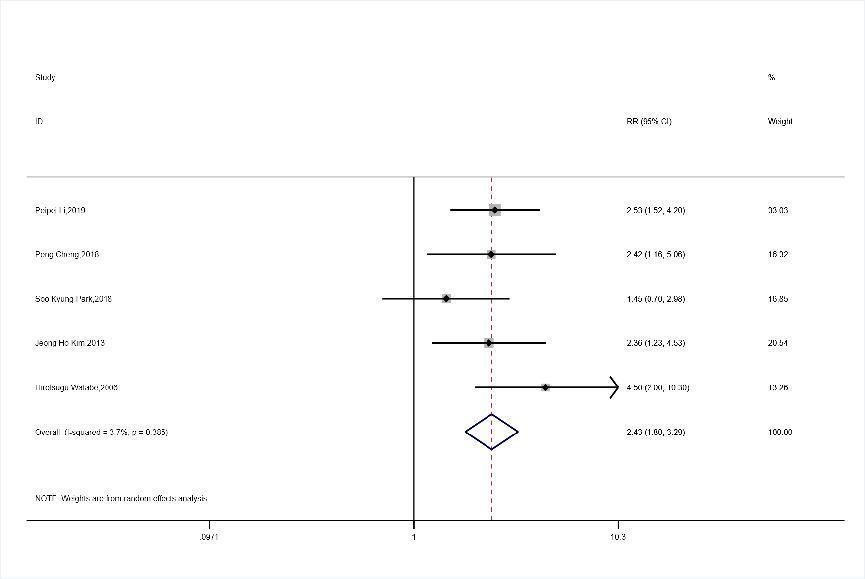


**Fig. S17.** Forest plot of polyp size ≥ 10 mm.

**
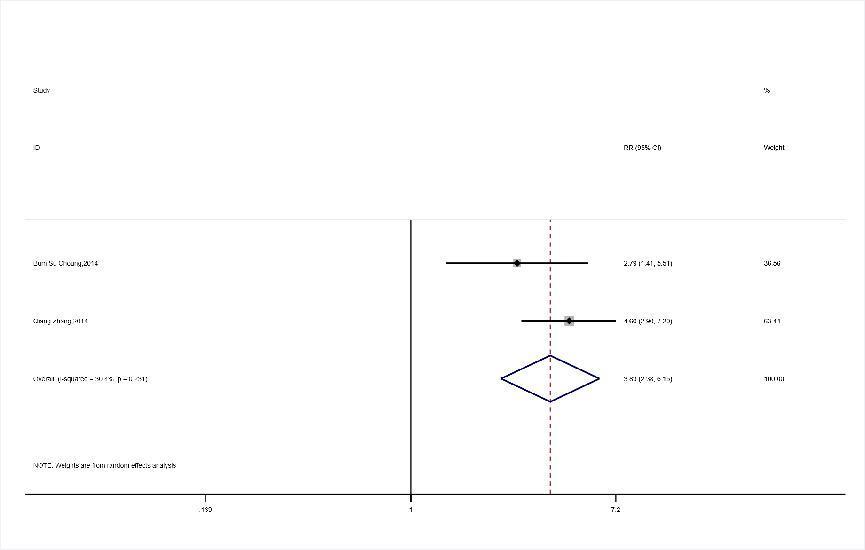
**

**Fig. S18.** Forest plot of polyp size > 10 mm.


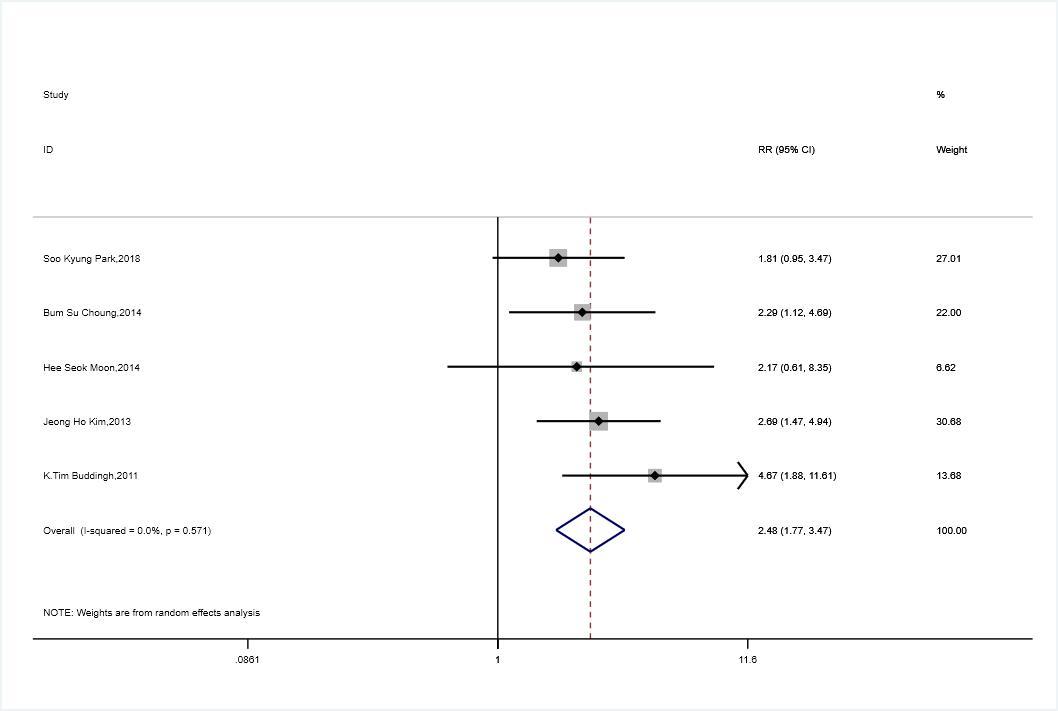


**Fig. S19.** Forest plot of polyp located in the right semicolon**.**

**
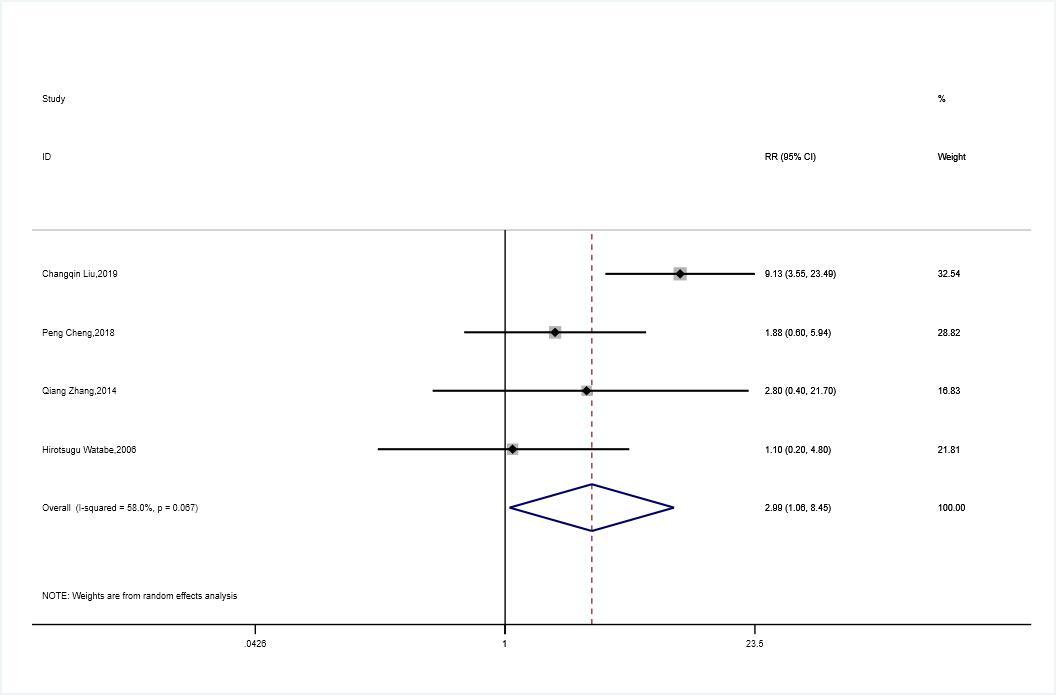
**

**Fig. S20.** Forest plot of endoscopic mucosal resection (EMR).

**
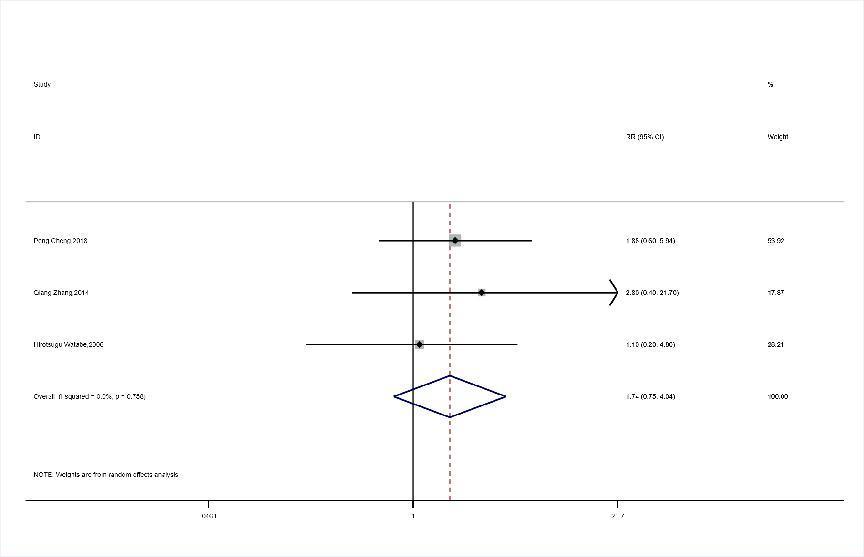
**

**Fig. S21.** Forest plot of endoscopic mucosal resection (EMR) after eliminate the article of Changqin Liu.
