# Supplementary material for: Risk factors for delayed colorectal postpolypectomy bleeding: a meta-analysis
Source: BMC Gastroenterol. 2024 May 14;24:162. doi: 10.1186/s12876-024-03251-6 (PMC11092041; doi:10.1186/s12876-024-03251-6)
Supplement: Supplementary file 4 — Supplementary Material 4 [file 12876_2024_3251_MOESM4_ESM.docx]

**Supplementary material 2** Search strategy.

1. **PubMed**

****((("Polyps"[Mesh]) OR ((polyps) OR (polyp))) AND (((((((blood loss, postoperative) OR (Postoperative Hemorrhages)) OR (Blood Loss, Postoperative)) OR (Loss, Postoperative Blood)) OR (Postoperative Blood Loss)) OR (Hemorrhages, Postoperative)) OR (Hemorrhage, Postoperative))) AND (((((((((((((((((((Risk Factors) OR (Factor, Risk)) OR (Risk Factor)) OR (Social Risk Factors)) OR (Factor, Social Risk)) OR (Factors, Social Risk)) OR (Risk Factor, Social)) OR (Risk Factors, Social)) OR (Social Risk Factor)) OR (Health Correlates)) OR (Correlates, Health)) OR (Population at Risk)) OR (Populations at Risk)) OR (Risk Scores)) OR (Risk Score)) OR (Score, Risk)) OR (Risk Factor Scores)) OR (Risk Factor Score)) OR (Score, Risk Factor))****

**2. Embase**

| No. | Query | Results | Date |
| --- | --- | --- | --- |
| #13 | #4 AND #8 AND #12 | 152 | 27-Nov-22 |
| #12 | #9 OR #10 OR #11 | 1642715 | 27-Nov-22 |
| #11 | 'risk factors':ab,ti | 793799 | 27-Nov-22 |
| #10 | 'risk factor':ab,ti | 371485 | 27-Nov-22 |
| #9 | 'risk factor'/exp | 1265180 | 27-Nov-22 |
| #8 | #5 OR #6 OR #7 | 46973 | 27-Nov-22 |
| #7 | 'blood loss, postoperative':ab,ti | 1660 | 27-Nov-22 |
| #6 | 'postoperative hemorrhage':ab,ti | 2330 | 27-Nov-22 |
| #5 | 'postoperative hemorrhage'/exp | 44780 | 27-Nov-22 |
| #4 | #1 OR #2 OR #3 | 104792 | 27-Nov-22 |
| #3 | 'polyps':ab,ti | 49185 | 27-Nov-22 |
| #2 | 'polyp':ab,ti | 32091 | 27-Nov-22 |
| #1 | polyp'/exp | 91876 | 27-Nov-22 |

**3. Cochrane**

ID Search Hits

#1 MeSH descriptor: [Colonic Polyps] explode all trees 580

#2 (Colonic Polyps):ti,ab,kw OR (Colonic Polyp):ti,ab,kw OR (Polyp, Colonic):ti,ab,kw OR (Polyps, Colonic):ti,ab,kw 1187

#3 #1 or #2 1187

#4 MeSH descriptor: [Postoperative Hemorrhage] explode all trees 1415

#5 (blood loss, postoperative):ti,ab,kw OR (Postoperative Hemorrhage):ti,ab,kw OR (Hemorrhages, Postoperative):ti,ab,kw OR (Postoperative Hemorrhages):ti,ab,kw OR (Postoperative Blood Loss):ti,ab,kw 14350

#6 (Loss, Postoperative Blood):ti,ab,kw OR (Hemorrhage, Postoperative):ti,ab,kw 14305

#7 #4 or #5 or #6 14362

#8 MeSH descriptor: [Risk Factors] explode all trees 26394

#9 (Risk Factors):ti,ab,kw OR (Risk Factor):ti,ab,kw OR (Factor, Risk):ti,ab,kw OR (Health Correlates):ti,ab,kw OR (Correlates, Health):ti,ab,kw 107898

#10 (Social Risk Factors):ti,ab,kw OR (Social Risk Factor):ti,ab,kw OR (Factors, Social Risk):ti,ab,kw OR (Risk Factor, Social):ti,ab,kw OR (Factor, Social Risk):ti,ab,kw 6019

#11 (Risk Factors, Social):ti,ab,kw OR (Populations at Risk):ti,ab,kw OR (Population at Risk):ti,ab,kw OR (Score, Risk Factor):ti,ab,kw OR (Score, Risk):ti,ab,kw 79778

#12 (Risk Factor Score):ti,ab,kw OR (Risk Score):ti,ab,kw OR (Risk Scores):ti,ab,kw OR (Risk Factor Scores):ti,ab,kw 45692

#13 #8 or #9 or #10 or #11 or #12 158688

#14 #3 and #7 and #13 16

**4. WOS**

TS=(Polyps) OR TS=(Polyp)

TS=(blood loss, postoperative) OR TS=(Postoperative Hemorrhages) OR TS=(Blood Loss, Postoperative) OR TS=(Loss, Postoperative Blood) OR TS=(Postoperative Blood Loss) OR TS=(Hemorrhages, Postoperative) OR TS=(Hemorrhage, Postoperative)

TS=(Risk Factors) OR TS=(Factor, Risk) OR TS=(Risk Factor) OR TS=(Social Risk Factors) OR TS=(Factor, Social Risk) OR TS=(Factors, Social Risk) OR TS=(Risk Factor, Social) OR TS=(Risk Factors, Social) OR TS=(Social Risk Factor) OR TS=(Health Correlates) OR TS=(Correlates, Health) OR TS=(Population at Risk) OR TS=(Populations at Risk) OR TS=(Risk Scores) OR TS=(Risk Score) OR TS=(Score, Risk) OR TS=(Risk Factor Scores) OR TS=(Risk Factor Score) OR TS=(Score, Risk Factor)
